# p300 Degradation by the p53‐SIAH1 Axis Relieves TBK1 Acetylation to Enhance Innate Antiviral Immunity

**DOI:** 10.1002/advs.76101

**Published:** 2026-06-15

**Authors:** Huidi Yu, Zhihao Zhan, Xiaoxiang Pan, Xinyue Zhang, Penggang Liu, Jing Sun, Xiulong Xu

**Affiliations:** ^1^ College of Veterinary Medicine Institute of Comparative Medicine Yangzhou University Yangzhou Jiangsu China; ^2^ College of Bioscience and Biotechnology Yangzhou University Yangzhou Jiangsu China; ^3^ Jiangsu Co‐innovation Center For Prevention and Control of Important Animal Infectious Diseases and Zoonosis Yangzhou University Yangzhou Jiangsu China

**Keywords:** Innate immunity, p300, p53, SIAH1, TBK1

## Abstract

p300 is an acetyltransferase that regulates gene expression by acetylating histones and transactivating some transcription factors such as nuclear Factor Kappa B (NF‐κB) and interferon regulatory factor 3 (IRF3). p53 is an interferon (IFN)‐inducible tumor suppressor that enhances antiviral responses. How p300 and p53 precisely regulate innate antiviral immunity remains incompletely understood. Herein, we report that conditional p300 knockout in alveolar epithelial cells does not suppress but rather enhances antiviral responses in mice infected with vesicular stomatitis virus (VSV) and herpes simplex virus (HSV‐1). In vitro investigation reveals that A‐485, a p300‐specific inhibitor, and p300 knockdown suppress virus replication but promote IFN‐β production in a variety of cell types by enhancing (TANK‐binding kinase 1) TBK1 and IRF3 phosphorylation. p300 binds TBK1 and acetylates two lysine residues at 241 and 692 to block its activation. p300 expression is downregulated by viral infection in a p53‐dependent manner. Mechanistically, viral infection increases the levels of p53, which leads to the upregulation of the seven in absentia homolog 1 (SIAH1) E3 ubiquitin ligase. SIAH1 induces p300 K48‐linked polyubiquitination and subsequent proteasomal degradation. Consistently, p53 knockout inhibits, whereas SIAH overexpression enhances antiviral responses. Taken together, our study identifies p300 as an acetyltransferase that suppresses innate immunity by acetylating TBK1, and demonstrates that the p53‐SIAH1 axis downregulates p300 to sustain antiviral responses.

## Graphical abstract

This study identifies p300 as the acetyltransferase that acetylates TBK1 and inhibits its phosphorylation. Activation of the p53‐SIAH1 axis by immune response downregulates p300 expression to sustain innate antiviral immunity. Conditional p300 knockout in alveolar epithelial cells in vivo promotes antiviral responses and suppresses virus replication.

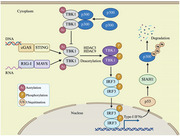

## 1 Introduction

Pattern recognition receptors (PRRs), including Toll‐like receptors (TLRs), RIG‐I （Retinoic acid‐inducible gene I ）‐like receptors (RLRs), and cytosolic DNA sensors, are central to initiating innate immune and inflammatory responses [1]. TLR3 and RLRs (RIG‐I and Melanoma differentiation‐associated gene 5，MDA5) detect extracellular and intracellular viral RNA, respectively [1]. RIG‐I specifically recognizes short dsRNA or 5’‐diphosphate/triphosphate dsRNA from viruses such as Sendai virus (SeV), VSV, and Newcastle disease virus (NDV) [2, 3]. Upon RNA binding, RIG‐I or MDA5 recruits the mitochondrial antiviral‐signaling protein (MAVS), which oligomerizes into prion‐like filaments [2, 3]. The MAVS aggregate then acts as a platform to activate TANK‐binding kinase 1 (TBK1) and interferon regulatory factor 3 (IRF3) [2, 3]. Similarly, cytosolic DNA is sensed by cyclic GMP‐AMP synthase (cGAS), which produces the second messenger 2’3’‐cGAMP [4, 5]. cGAMP binds to the endoplasmic reticulum protein STING, triggering its aggregation and translocation to the Golgi complex, where it recruits and activates TBK1 to phosphorylate IRF3 [4, 5, 6, 7, 8]. Phosphorylated IRF3 dimerizes, enters the nucleus, and drives transcription of type I interferon genes [6, 7, 8].

TBK1 activity is modulated by post‐translational modifications such as phosphorylation, ubiquitination, acetylation, and sumoylation [9, 10]. While histone deacetylase 3 (HDAC3) and HDAC9 can deacetylate TBK1 and promote its activation [11, 12], the acetyltransferase responsible for acetylating TBK1 has not been identified [9, 10]. p300 is a histone acetyltransferase that primarily functions to acetylate histones H3 and H4 (H3K27ac) and various transcription factors, including p53, c‐Myc, GALA‐1, and NF‐κB, thereby influencing their activity [13, 14]. p300 is recruited and activated by dimerized IRF3 and (Signal Transducer And Activator Of Transcription 1 (STAT1 [15, 16]. Although viral infection increases acetylation of histones at the *Ifnb* promoter, direct evidence that p300 enhances IFN production or innate immunity remains rare [17]. In fact, recent work by Zeng et al. [18] indicates that p300 deficiency elevates IFN‐β and major histocompatibility complex class I (MHC‐I) expression and improves antigen presentation, raising uncertainty about the role of p300 in innate and adaptive immunity.

The tumor suppressor p53 regulates diverse cellular processes, including cell division, apoptosis, senescence, and metabolism [19, 20]. It also transcriptionally controls immune‐related genes such as *ISG15*, *IRF9*, *IRF7*, *IRF5*, *STING1*, and *TLR3* [21, 22]. p53‐deficient cells are more permissive for Sendai and influenza virus replication in vitro and in vivo [23, 24], in part due to p53‐mediated apoptosis and increased IFN production [23, 24, 25]. p53 expression itself is induced by type I IFN signaling, suggesting a positive feedback loop during antiviral responses [22, 26]. SIAH1, an E3 ubiquitin ligase induced by p53, restricts viral replication by ubiquitinating viral proteins and targeting USP19 to prevent the de‐ubiquitination of MAVS, TNF Receptor Associated Factor 3 (TRAF3), and TIR domain‐containing adapter‐inducing IFN‐β (TRIF) [27, 28]. How p53 and SIAH1 regulate innate immunity to restrict virus replication remains unclear.

In the present study, we demonstrate that pharmacological or genetic inhibition of p300 enhanced IFN gene expression. Mechanistically, p300 acetylated TBK1 and suppressed its activation. p300 expression was downregulated via SIAH1‐mediated polyubiquitination and proteasomal degradation. p53 knockout abrogated SIAH1 expression, stabilized p300, attenuated antiviral immunity, and increased viral replication. In vivo, conditional knockout of p300 in alveolar epithelial cells strengthened local antiviral immunity and protected mice from lethal VSV infection. Together, these findings indicate that p300 promotes viral replication by acetylating TBK1 to dampen innate immunity, and that the p53–SIAH1 axis sustains antiviral responses by targeting p300 for degradation.

## 2 Results

### 2.1 p300 Deficiency Enhances Host Defense and Protects Mice From Viral Infection

**FIGURE 1:**
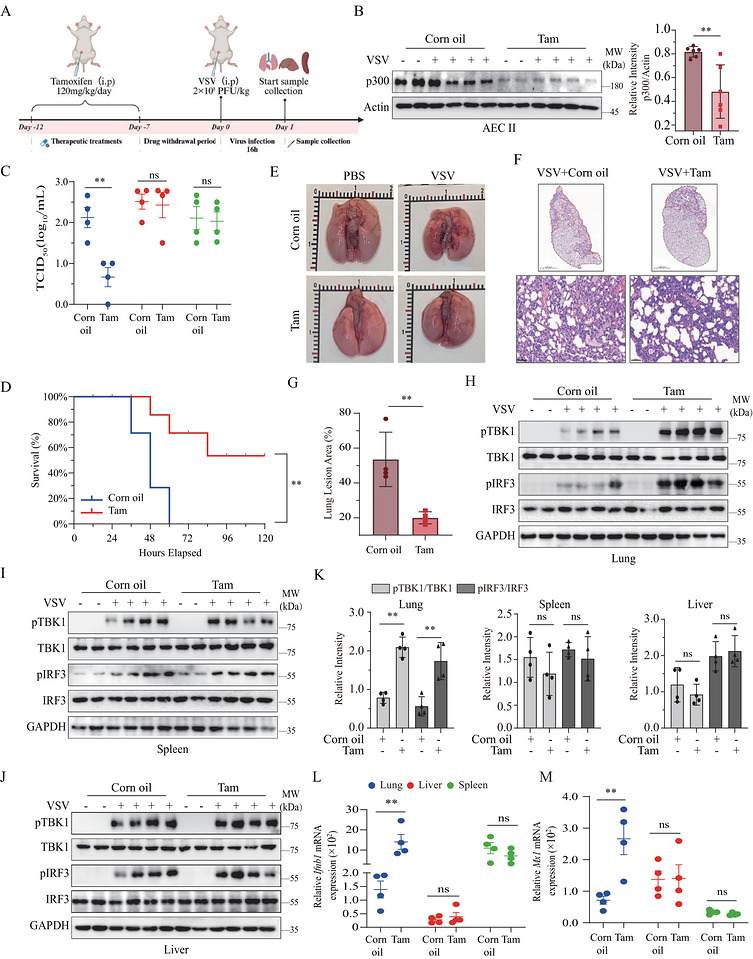
p300 deficiency boosts antiviral responses in vivo. (A, B) Induction of conditional p300 knockout (cKO) and virus infection scheme. p300^flox/flox^ × Sftpc‐CreER^T2^ mice were administered intraperitoneally (i.p.) with corn oil as the vehicle control or with Tamoxifen (120 mg/kg/d) dissolved in corn oil for 1 week to induce p300 conditional knockout in the pulmonary epithelial cells. Control and p300 cKO mice (Male, 6–7‐weeks‐old) were left uninfected (2 mice per group) or infected with VSV (2 × 10^8^ PFU/kg) (4 mice per group) by intraperitoneal infection. Mice were euthanized 16 h post‐infection. Alveolar epithelial cells isolated from a portion of the lung tissue were assessed for p300 expression by Western blot. (C) p300 deficiency inhibits VSV replication. Tissue lysates homogenized from the second portion of the lung, liver, and spleen in PBS were analyzed for viral loads by assaying TCID_50_. Data are presented as the mean ± standard deviation (SD) of four mice each. An unpaired Student's *t*‐test was used to determine the statistical significance. ^**^ *p* < 0.01; ns, not significant. (D) p300 deficiency improves the survival of VSV‐infected mice. Control and p300 cKO male mice (10 mice per group, male) were intraperitoneally injected with VSV (2 × 10^9^ PFU/kg). Survival was monitored daily and statistically analyzed by the Kaplan‐Meier method with the Log‐Rank test. ^**^ *p* < 0.01. (E–G) Gross and histological changes in the lungs. Gross pathological changes in the lungs from uninfected and VSV‐infected control and p300 cKO mice (4 mice per group) (E). The sections of lung tissues were stained with hematoxylin and eosin (H&E) (F) followed by quantification of the inflammation area (G). Data are presented as the mean ± SD of four mice each. An unpaired Student's *t*‐test was used to determine the statistical significance. ^**^ *p* < 0.01. (H–K) p300 deficiency enhances TBK1 and IRF3 phosphorylation. Lung, liver, and spleen tissue lysates were analyzed for TBK1 and IRF3 phosphorylation by Western blot (H‐J). Relative TBK1 and IRF3 phosphorylation in the tissue lysates from four VSV‐infected mice were analyzed by using Image‐J, normalized to their corresponding total proteins (K). Data are presented as the mean ± SD of four mice each. An unpaired Student's *t*‐test was used to determine the statistical significance. ^**^ *p* < 0.01. ns, not significant. (L, M) p300 deficiency enhances *Ifnb1* and *Mx1* mRNA transcription. Total tissue RNA extracted from the third portion of the lung, liver, and spleen of VSV‐infected mice was analyzed for the levels of *Ifnb1* (L) and *Mx1* (M) using RT‐PCR. Data represent the mean ± SD from 4 samples each. An unpaired Student's *t*‐test was used to determine the statistical significance. ^*^ *p* < 0.05, ^**^ *p* < 0.01.

p300 has been implicated in enhancing antiviral responses by engaging IRF3 and NF‐κB to promote IFN gene transcription [13, 14, 29]. We first tested if p300 knockout would inhibit antiviral responses to promote virus replication in vivo. We generated p300 conditional knockout (cKO) mice in which the *p300* gene was specifically knocked out in type II lung epithelial cells by treating p300^flox/flox^ × Sftpc‐CreER^T2^ mice with Tamoxifen (Tam) (Figure 1A). Western blot analysis revealed that p300 expression was significantly downregulated in lung epithelial cells isolated from the lungs of Tam‐treated mice (Figure 1B). Unexpectedly, virus titers were substantially lower in the lungs of Tam‐treated mice infected with VSV (Figure 1C) or HSV‐1 (Figure S1A) than in the corn oil‐treated control mice. In contrast, viral loads were not significantly different in the livers and spleens of Tam‐treated mice and vehicle‐treated control mice infected with VSV (Figure 1C). *p300* cKO mice infected with VSV survived significantly longer than wild‐type mice (Figure 1D). Gross examination of pathological changes revealed that VSV infection induced severe pulmonary congestion with a heavily vascularized, dark red surface in the lungs of vehicle‐treated control mice but not in those from Tam‐treated mice (Figure 1E). Hematoxylin and Eosin (H & E) staining of the lung sections revealed that VSV infection caused severe pulmonary hemorrhage in the lungs of vehicle‐treated control mice (Figure 1F). By contrast, VSV infection caused heavy immune cell infiltration in the lung tissue of Tam‐treated mice (Figure 1F). The areas of inflammatory legions were significantly larger in the lungs of VSV‐infected wild‐type mice than in those of VSV‐infected *p300* cKO mice (Figure 1G).

We then tested if p300 deficiency affected innate immunity in vivo in the tissues of the lungs, spleen, and liver. p300 deficiency in alveolar epithelial cells enhanced TBK1 and IRF3 phosphorylation in the lung tissues of VSV‐infected mice (Figure 1H,K) and HSV‐1‐infected mice (Figure S1B,C) but not in liver or spleen tissues of VSV‐infected mice (Figure 1I–K). RT‐PCR analysis revealed that the levels of *Ifnb1* and *Mx1* mRNA were significantly increased in the lungs of cKO mice compared to the lungs of wild‐type mice infected with VSV (Figure 1L,M) or HSV‐1 (Figure S1D,E). However, there were no significant differences in the levels of *Ifnb1* and *Mx1* mRNA in the liver or spleen tissues of corn oil‐ and Tam‐treated mice infected with VSV (Figure 1L,M). These observations collectively suggest that conditional p300 knockout in type II lung epithelial cells does not inhibit but rather enhances lung‐specific antiviral immunity, resulting in the restriction of VSV and HSV‐1 replication and improvement in the survival of VSV‐infected mice.

### 2.2 p300 Facilitates Virus Replication by Suppressing Antiviral Responses

**FIGURE 2:**
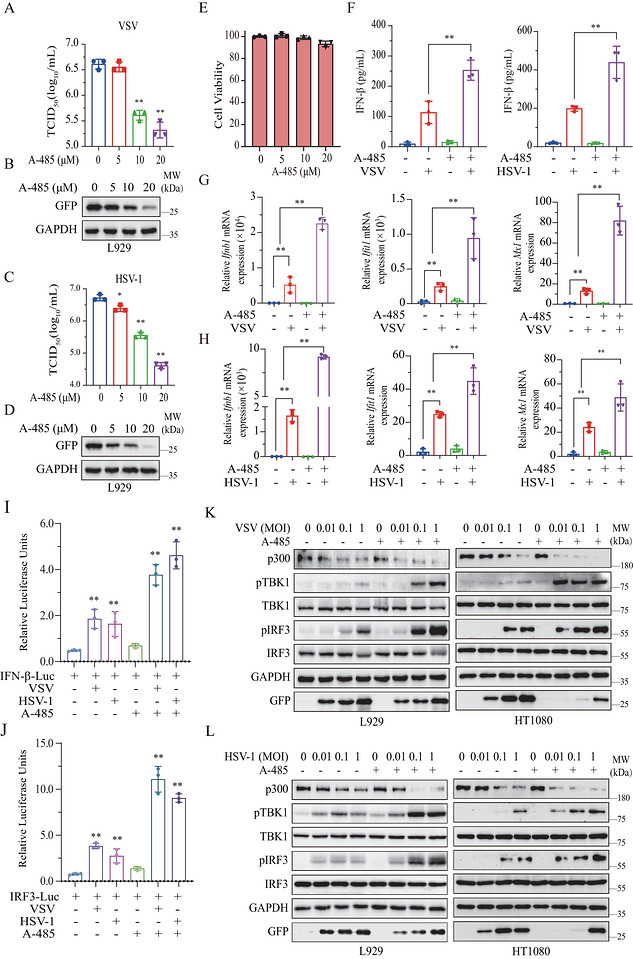
p300 inhibition enhances innate immunity and suppresses viral replication. (A–D) A‐485 inhibits virus replication. L929 cells pre‐treated with the indicated concentrations of A‐485 (0, 5, 10, and 20 µm) for 16 h were infected with 0.02 MOI of VSV (A, B) or HSV‐1 (C, D). After incubation for 16 h, the conditioned media were collected and analyzed for virus titers by measuring the TCID_50_ values (A, C). Data are presented as the mean ± SD of three independent experiments in bar graphs. An unpaired Student's *t*‐test was used to determine the statistical significance. ^*^ *p* < 0.05, ^**^ *p* < 0.01. Cell lysates were prepared and analyzed for GFP reporter expression by probing with an antibody against GFP (B, D). (E) A‐485 lacks cytotoxicity on L929 cells. L929 cells seeded in a 96‐well plate were incubated in the absence or presence of the indicated concentrations of A‐485 for 48 h. Cytotoxicity was measured by using a CCK‐8 kit. Data are presented as the mean ± SD of triplicate measurements from one of three experiments with similar results. (F–H) A‐485 enhances IFN‐β production. L929 cells were incubated in the absence or presence of A‐485 (5 µm) for 6 h and then left uninfected or infected with VSV or HSV‐1 (1 MOI each). After incubation for 12 h, conditioned media were collected and assayed for IFN‐β levels by ELISA (F). Total cellular RNA was extracted and analyzed for *Ifnb1*, *Ifit1*, and *Mx1* mRNA levels by RT‐PCR (G, H). Data represent the mean ± SD from three independent experiments. An unpaired Student's *t*‐test was used to determine the statistical significance. ^**^p < 0.01. (I, J) A‐485 enhances IFN‐β and IRF3 binding site‐driven luciferase expression. 293T cells were transfected with the IFN‐β promoter or IRF3‐binding site‐driven luciferase reporter plasmids and incubated for 24 h. Cells were incubated in the absence or presence of A‐485 (5 µm for VSV, 10 µm for HSV‐1) for 6 h and then infected with VSV or HSV‐1 (1 MOI each). After incubation for 12 h, cell lysates were prepared and analyzed for luciferase activity. Data represent the mean ± SD of triplicate measurements from one of three independent experiments with similar results. An unpaired Student's *t*‐test was used to determine the statistical significance. ^**^p < 0.01. (K, L) A‐485 enhances TBK1 and IRF3 phosphorylation. L929 and HT1080 cells were incubated in the absence or presence of A‐485 (5 µm) for 6 h and then left uninfected or infected with the indicated MOIs of VSV or HSV‐1 for 12 h. Cell lysates were prepared and analyzed for the indicated proteins by Western blot. Representative blots from one of three independent experiments with similar results are shown.

C646, a p300‐specific inhibitor, attenuates influenza virus replication [30]. We then tested if pharmacologic inhibition of p300 could restrict VSV and HSV‐1 replication by enhancing innate immunity. A‐485, a p300‐specific inhibitor [31], dose‐dependently lowered the titers of VSV and HSV‐1 in L929 cells (Figure 2A,C) and inhibited the expression of the green fluorescence protein (GFP) reporter protein (Figure 2B,D) at 16 h post‐infection. Similar observations were made in L929 cells infected with SeV (Figure S2A,B). Inhibition of virus replication by A‐485 was not due to its cytotoxicity since A‐485 used at these concentrations did not affect L929 cell viability (Figure 2E). Consistently, A‐485 at 10 µm also effectively inhibited GFP expression in RAW264.7 cells (a murine macrophage cell line) (Figure S3A–F) and NL20 cells (a human bronchial epithelial cell line) infected with VSV or HSV‐1 (Figure S3G–L). A‐485 treatment significantly increased the levels of IFN‐β in the conditioned media of L929 (Figure 2F), RAW264.7 (Figure S3M,N), and NL20 cells (Figure S3O,P) infected with VSV or HSV‐1. Consistently, A‐485 treatment significantly heightened the levels of *Ifnb1, Ifit1*, and *Mx1* mRNA in L929 cells infected with VSV or HSV‐1 (Figure 2G,H). A‐485 also enhanced the *Ifnb1* promoter‐ or IRF3 binding site‐driven luciferase expression in L929 cells infected with VSV or HSV‐1 (Figure 2I,J). Mechanistically, A485 enhanced TBK1 and IRF3 phosphorylation in L929 and HT1080 cells (Figure 2K,L), RAW264.7 (Figure S3A,D), and NL20 cells (Figure S3G,J) infected with VSV or HSV‐1. Notably, virus infection also dose‐dependently decreased p300 expression in these cell lines (Figure 2K,L; Figure S3A,D,F,G). A‐485 combined with virus infection further downregulated p300 expression (Figure 2K,L; Figure S3A,D,F,G). A‐485 also decreased the levels of the *GFP* reporter gene expression (Figure 2K,L; Figure S3A,D,F,G). A‐485 also enhanced TBK1 and IRF3 phosphorylation in L929 and HT1080 cells infected with SeV (Figure S2C,D). These observations collectively suggest that inhibition of p300 activity by A‐485 promotes innate antiviral immunity to suppress the replication of both RNA and DNA viruses.

### 2.3 p300 Suppresses Innate Immunity

**FIGURE 3:**
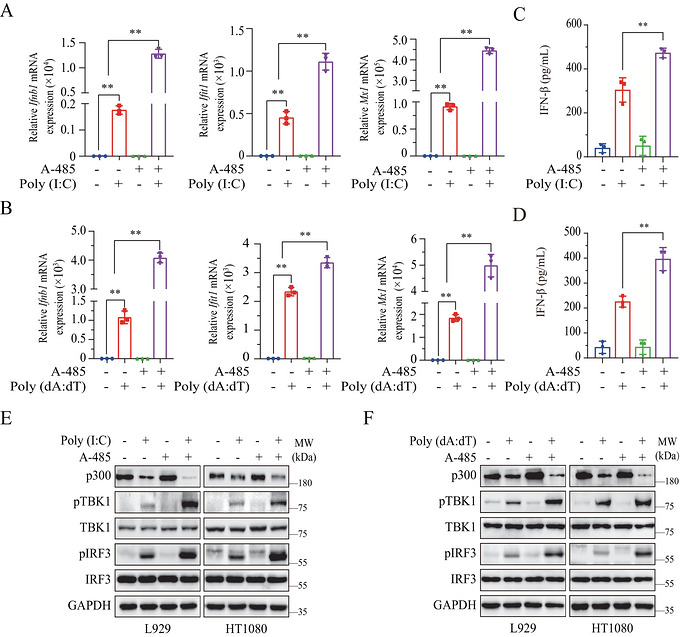
p300 inhibition enhances poly (I:C)‐ and poly (dA:dT)‐induced innate immunity. (A, B) A‐485 enhances IFN‐β and ISG gene transcription. L929 cells were incubated in the absence or presence of A‐485 (5 µm) for 6 h and then transfected with poly (I:C) (1 µg) or poly (dA:dT) (1 µg) and incubated for 8 h. The levels of *Ifnb1*, *Ifit1*, and *Mx1* mRNA were analyzed by RT‐PCR. The results represent the mean ± SD of three independent experiments. An unpaired Student's *t*‐test was used to determine the statistical significance. ^**^ *p* < 0.01. (C, D) A‐485 enhances IFN‐β production. L929 cells were incubated in the absence or presence of A‐485 (5 µm) for 6 h and then transfected with poly (I:C) (1 µg) or poly (dA:dT) (1 µg). After incubation for 8 h, conditioned media were collected and assayed for IFN‐β levels by ELISA. Data represent the mean ± SD from three independent experiments. An unpaired Student's *t*‐test was used to determine the statistical significance. ^**^ *p* < 0.01. (E, F) A‐485 enhances TBK1 and IRF3 phosphorylation. L929 and HT1080 cells were incubated in the absence or presence of A‐485 (5 µm) for 6 h and then transfected with poly (I:C) (1 µg) or poly (dA:dT) (1 µg) and incubated for 8 h. Cell lysates were prepared and analyzed for the levels of p300 and TBK1 and IRF3 phosphorylation by Western blot. Representative blots from one of three independent experiments with similar results are shown.

We next tested whether A‐485 also enhanced innate immunity in L929 cells activated by nucleic acid sensors. A‐485 treatment further increased the levels of *Ifnb1, Ifit1*, and *Mx1* mRNA in L929 cells transfected with poly (I:C) or poly (dA:dT) (Figure 3A,B). A‐485 increased the levels of IFN‐β in the conditioned media of poly (I:C)‐ or poly (dA:dT)‐stimulated L929 cells (Figure 3C,D) and enhanced TBK1 and IRF3 phosphorylation in poly (I:C)‐ and poly (dA:dT)‐stimulated L929 and HT1080 cells (Figure 3E,F). Of note, p300 expression was reduced in both cell lines transfected with poly (I:C) or poly (dA:dT) (Figure 3E,F). A‐485 treatment further decreased the levels of p300 expression (Figure 3E,F), which is likely due to the further activation of the immune response that downregulates p300 expression by the p53‐SIAH1 axis as shown below. These observations collectively suggest that p300 inhibition enhances innate immunity, and that activation of innate immunity downregulates p300 expression.

### 2.4 p300 Knockdown Restricts Virus Replication and Enhances Innate Antiviral Immunity

**FIGURE 4:**
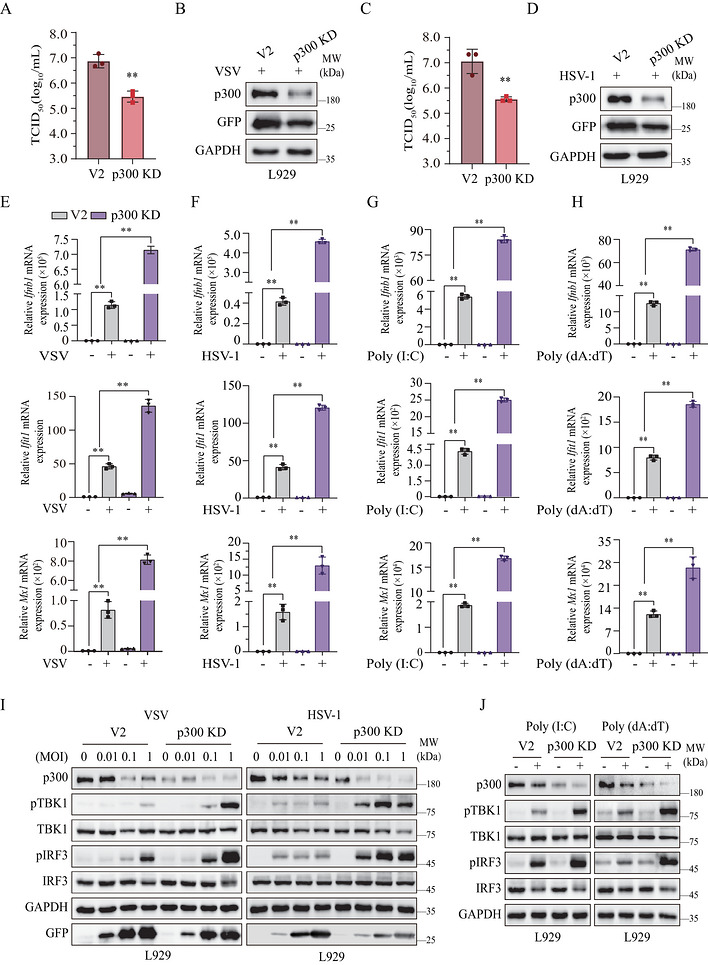
p300 silencing enhances innate immunity and suppresses virus replication. (A–D) p300 silencing inhibits VSV and HSV‐1 replication. Control pLentiCRISPRv2 (V2) and p300 knockdown (p300 KD) L929 cells were infected with VSV or HSV‐1 (0.02 MOI each). After incubation for 16 h, conditioned media were collected and analyzed for virus titer (A, C). Cell lysates were analyzed for p300 and GFP expression by Western blot (B, D). (E, F) p300 knockdown lowers virus‐induced IFN‐β and ISG mRNA levels. Control (V2) and p300 KD L929 cells were infected with VSV and HSV‐1 (1 MOI each) and incubated for 12 h. The levels of *Ifnb1*, *Ifit1*, and *Mx1* mRNA were analyzed by RT‐PCR. (G, H) p300 knockdown lowers poly (I:C) and poly (dA:dT)‐induced IFN‐β and ISG mRNA levels. Control (V2) and p300 KD L929 cells were transfected with poly (dA:dT) (1 µg) or poly (I:C) (1 µg). After incubation for 12 h, the levels of *Ifnb1*, *Ifit1*, and *Mx1* mRNA were analyzed by RT‐PCR. (A–H) Data represent the mean ± SD from three independent experiments. An unpaired Student's *t*‐test was used to determine the statistical significance. ^**^ *p* < 0.01. (I) p300 knockdown inhibits virus‐induced TBK1 and IRF3 phosphorylation. Control (V2) and p300 KD L929 cells were left uninfected or infected with the indicated MOIs of VSV or HSV‐1. After incubation for 12 h, cell lysates were prepared and analyzed for the indicated proteins by Western blots. (J) p300 knockdown enhances poly (I:C)‐ or poly (dA:dT)‐induced TBK1 and IRF3 phosphorylation. Control (V2) and p300 KD L929 cells were transfected with poly (I:C) (1 µg) or poly (dA:dT) (1 µg). After incubation for 8 h, cell lysates were prepared and analyzed for the expression of the indicated proteins by Western blots. (I, J) Representative blots from one of three independent experiments with similar results are shown.

To determine the specificity of A‐485‐mediated suppression on innate immunity, we then tested if p300 knockdown (KD) would have the same effects. p300 knockdown lowered the titers of VSV and HSV‐1 in the conditioned media of L929 cells and reduced the *GFP* reporter gene expression in cell lysates at 16 h post infection (Figure 4A–D). Similar observations were made in L929 cells infected with SeV (Figure S2E,F). Quantitative RT‐PCR analysis revealed that p300 knockdown increased the levels of *Ifnb1, Ifit1*, and *Mx1* mRNA in L929 cells infected with VSV and HSV‐1 (Figure 4E,F) or SeV (Figure S2G) or stimulated by poly (I:C) or poly (dA:dT) (Figure 4G,H). p300 knockdown also enhanced TBK1 and IRF3 phosphorylation in L929 cells infected with VSV, HSV‐1 (Figure 4I), or SeV (Figure S2H) or stimulated by poly (I:C) or poly (dA:dT) (Figure 4J). Again, both virus infection and cytosolic nucleic acids decreased p300 expression (Figure 4I,J).

p300 overexpression blocked TBK1 and IRF3 phosphorylation in 293T cells transfected with poly (I:C) (Figure S4A) or infected with SeV or HSV‐1 (Figure S4B,C). Consistently, p300 overexpression lowered the levels of *IFNB1, IFIT1*, and *MX1* mRNA in 293T cells stimulated with poly (I:C) (Figure S4D) or infected with SeV (Figure S4E) or HSV‑1 (Figure S4F). Luciferase reporter assays revealed that p300 overexpression markedly inhibited the IRF3 binding site‐ and *Ifnb* promoter‐driven luciferase reporter gene expression in 293T cells infected with SeV or VSV (Figure S4G,H). Collectively, these observations suggest that p300 antagonizes innate immunity.

### 2.5 p300 Acetylates TBK1 at K241 and K692 to Inhibit Its Activation

**FIGURE 5:**
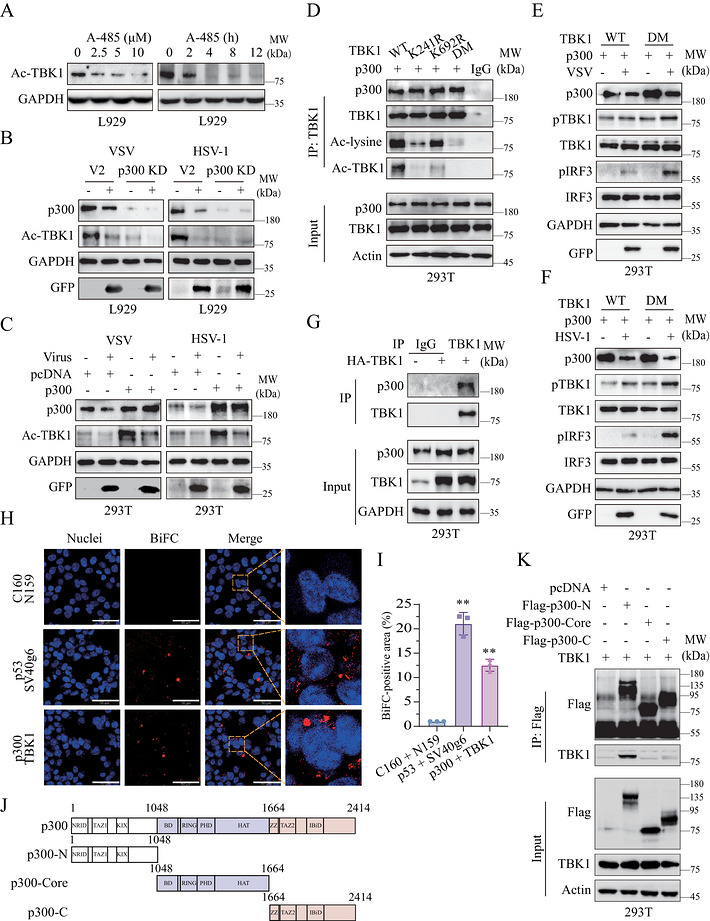
p300 binds and acetylates TBK1. (A) A‐485 inhibits TBK1^K241^ acetylation. L929 cells were treated with the indicated concentrations of A‐485 for 12 h or with 5 µm A‐485 for the indicated lengths of time. TBK1 acetylation was detected by Western blot with an Ac241‐specific antibody. (B) p300 knockdown inhibits TBK1 acetylation. Control (V2) and p300 KD L929 cells were left uninfected or infected with 1 MOI of VSV or HSV‐1 (1 MOI each) and incubated for 12 h. TBK1 acetylation was detected by Western blot with an Ac241‐specific antibody. (C) p300 overexpression induces TBK1 acetylation. 293T cells were transfected with pcDNA3.1 or a p300 expression vector for 36 h. Cells were then left uninfected or infected with VSV or HSV‐1 (1 MOI each) for 12 h. Cell lysates were prepared and analyzed for TBK1^K241^ acetylation by Western blot with an Ac241‐specific antibody. (D) p300 acetylates TBK1 at K241 and K692. 293T cells were transfected with the p300 expression vector plus the expression vector encoding wild‐type TBK1 (WT) or K241R and K692R mutant TBK1 or K241R/K692 double‐mutant TBK1 (DM). Cell lysates were immunoprecipitated with an anti‐TBK1 antibody followed by probing with an anti‐p300 and anti‐lysine‐acetyl antibody. (E, F) K241/K692 mutations render TBK1 resistant to p300 inhibition. 293T cells were transfected with p300 plus an expression vector encoding wild‐type or DM mutant TBK1. After incubation for 36 h, the cells were left uninfected or infected with VSV or HSV‐1 (1 MOI each) and then incubated for 12 h. Cell lysates were prepared and analyzed for p300 expression and TBK1 and IRF3 phosphorylation. (G) TBK1 binds p300. 293T cells were transfected with HA‐tagged TBK1 (HA‐TBK1) and incubated for 48 h. Cell lysates were immunoprecipitated with an anti‐TBK1 antibody or normal rabbit IgG as a control and then probed with antibodies against p300 and TBK1. Cell lysates (input) were analyzed for transfection efficiency with anti‐TBK1 and anti‐p300 antibodies. (H, I) In situ TBK1 and p300 interaction.293T cells seeded on the coverslips were co‐transfected with C160‐p300 plus N159‐TBK1 or C160 plus N159 as a negative control. C160‐p53 and N159‐SV40gp6 were included as a positive control. After incubation for 48 h, the protein‐protein interaction was examined under a fluorescence microscope. Nuclei were stained with DAPI (blue). Scale bar, 50 µm. Fluorescence intensity was quantified as the ratio of red fluorescence area to total cell area using ImageJ (NIH). Data are presented as the mean ± SD of three randomly selected fields from one of three independent experiments with similar results. An unpaired Student's *t*‐test was used to determine the statistical significance. *p* < 0.01.(J, K) Schematic illustration of the full‐length p300 and the N‐, Core‐, and C‐terminal truncations (J). 293T cells were co‐transfected with the HA‐TBK1 expression vector plus the expression vector encoding the truncated N‐, Core‐, or C‐terminal p300 (Flag–p300‐N, Flag–p300‐Core, or Flag–p300‐C). After incubation for 48 h, cell lysates were immunoprecipitated with an anti‐Flag antibody and then probed with an anti‐TBK1 and anti‐Flag antibody. (A–G, K) Representative blots from one of three independent experiments with similar results are shown.

TBK1 acetylation at Lys241 and Lys692 inhibits its phosphorylation and activation [11, 12]. Having shown the inhibitory effect of p300 on TBK1 phosphorylation, we hypothesized that p300 as an acetyltransferase may acetylate TBK1 and inhibit its activation. Using a K241‐specific antibody, we found that A‐485 treatment dose‐ and time‐dependently reduced TBK1 K241 acetylation (Figure 5A). VSV and HSV‐1 infection itself also decreased TBK1 K241 acetylation, likely due to decreased p300 expression (Figure 5B). p300 knockdown abrogated TBK1 K241 acetylation in uninfected and VSV‐ or HSV‐1‐infected L929 cells (Figure 5B). Conversely, p300 overexpression enhanced TBK1 K241 acetylation in uninfected or virus‐infected 293T cells (Figure 5C).

Acetylation at K241 and K692 in TBK1 is implicated in inhibiting TBK1 phosphorylation and activation [11, 12]. We then tested whether p300 was responsible for acetylating these two lysine residues. To do so, the lysine residue at 241 and 692 in TBK1 was individually or simultaneously substituted with an arginine residue to generate K241R, K692R, and K241R/K692R (DM) mutants. Wild‐type or mutant TBK1 co‐transfected with p300 in 293T cells was immunoprecipitated and detected with an anti‐Ac‐lysine antibody and a K241‐specific antibody. p300 overexpression increased wild‐type TBK1 acetylation more effectively than the K241R or K692R mutant TBK1 (Figure 5D) but could not acetylate DM‐TBK1 at all (Figure 5D). Mutation at these two lysine residues did not affect p300 binding to TBK1 (Figure 5D). We next investigated the impact of the TBK1 acetylation sites on its activation. VSV and HSV‐1 infection induced TBK1 and IRF3 phosphorylation poorly in 293T cells co‐transfected with p300 plus wild‐type TBK1 expression vectors but very effectively in those co‐transfected with p300 plus DM‐TBK1 (Figure 5E,F). These observations collectively suggest that p300 is the acetyltransferase responsible for acetylating TBK1 at K241 and K692.

p300 interacts with TBK1. We next evaluated the physical interaction between p300 and TBK1. Endogenous p300 in HA‐TBK1‐transfected 293T cells was brought down by an anti‐HA antibody (Figure 5G). Bimolecular fluorescence complementation (BiFC) assays revealed that p300 was co‐localized with TBK1 in the cytoplasm of 293T cells (Figure 5H,I). The p53‐SV40 gp6 interaction was included as a positive control. As reported in our prior study [32], the p53‐SV40 interaction forms red puncta seen in the cytoplasm (Figure 5H,I). In contrast, the empty vector included as a negative control did not illuminate at all (Figure 5H,I). To map the interaction domain of p300 with TBK1, we generated three Flag‐tagged p300 truncation constructs that cover the N‐, middle‐, and C‐terminal domains. Co‐immunoprecipitation experiments with an anti‐Flag antibody revealed that only the N‐terminal region brought down TBK1 (Figure 5J,K).

### 2.6 Virus Infection Induces p300 Proteasomal Degradation

**FIGURE 6:**
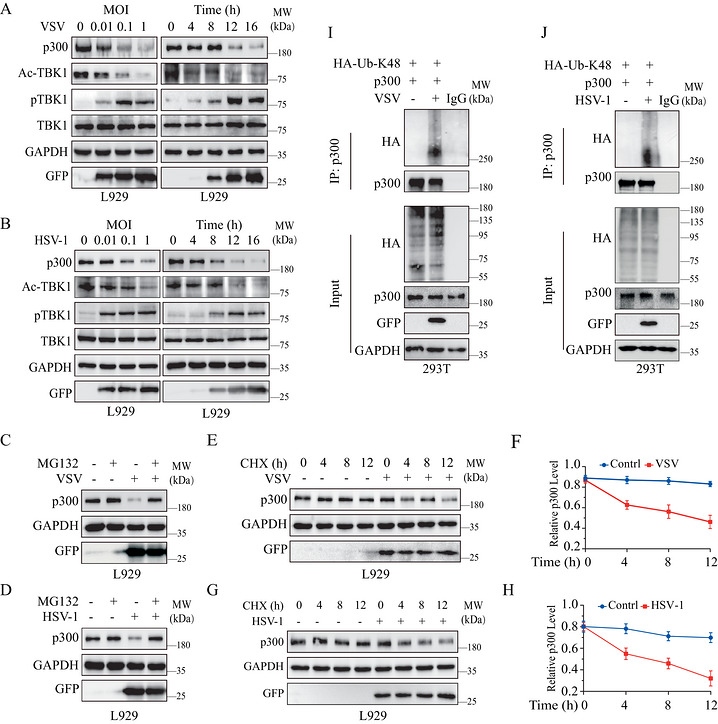
Virus infection decreases p300 expression via proteasomal degradation. (A, B) VSV infection reduces p300 expression and TBK1 acetylation. L929 cells were infected with the indicated MOIs of VSV (A) and HSV‐1 (B) for 12 h, or with 1 MOI of these viruses for the indicated lengths of time. Cell lysates were prepared and analyzed by Western blot for p300 expression, TBK1^K241^ acetylation, and TBK1 phosphorylation. (C, D) L929 cells were infected with 1 MOI of VSV or HSV‐1 for 8 h and then incubated in the absence or presence of MG132 (10 µm) for another 12 h. Cell lysates were analyzed for p300 expression by Western blot. GFP and GAPDH were detected as viral infection and loading controls. (E–H) Virus infection shortens p300 half‐life. L929 cells were infected with VSV (E, F) and HSV‐1 (G, H) (1 MOI each) for 4 h and then treated with CHX (100 µM) for the indicated lengths of time. Cell lysates were prepared and analyzed for p300 expression. Relative p300 levels were quantified and presented in a bar graph (F, H). Data represent the mean ± SD from three independent experiments. (I, J) Virus infection induces p300 K48 polyubiquitination. 293T cells were co‐transfected with p300 and HA‐tagged K48 ubiquitin (HA‐Ub‐K48) plasmids. After incubation for 36 h, the cells were left uninfected or infected with VSV and HSV‐1 (1 MOI each). After incubation in the presence of MG132 (10 µm) for 12 h, cell lysates were immunoprecipitated with an anti‐p300 antibody followed by detection of ubiquitination with an anti‐HA mAb. Cell lysates were analyzed for the expression of indicated proteins by Western blot as input controls. Representative blots from one of three independent experiments with similar results are shown.

Finally, we investigated the mechanisms by which virus infection induced p300 degradation. p300 expression was dose‐ and time‐dependently decreased in L929 and HT1080 cells infected with VSV or HSV‐1 (Figure 6A,B). TBK1 K241 acetylation was downregulated and corresponded with p300 levels (Figure 6A,B). Similar observations were made in L929 cells infected with SeV and in HT1080 cells infected with SeV, VSV, or HSV‐1 (Figure S5). The proteasomal inhibitor MG132 prevented VSV‐ and HSV‐1‐induced p300 degradation (Figure 6C,D). VSV and HSV‐1 infection decreased p300 half‐life (Figure 6E–H) and p300 K48 polyubiquitination (Figure 6I,J) in L929 cells. These observations suggest that virus infection downregulates p300 expression by proteasomal degradation.

### 2.7 Virus Infection Activates the p53‐SIAH1 to Downregulate p300

**FIGURE 7:**
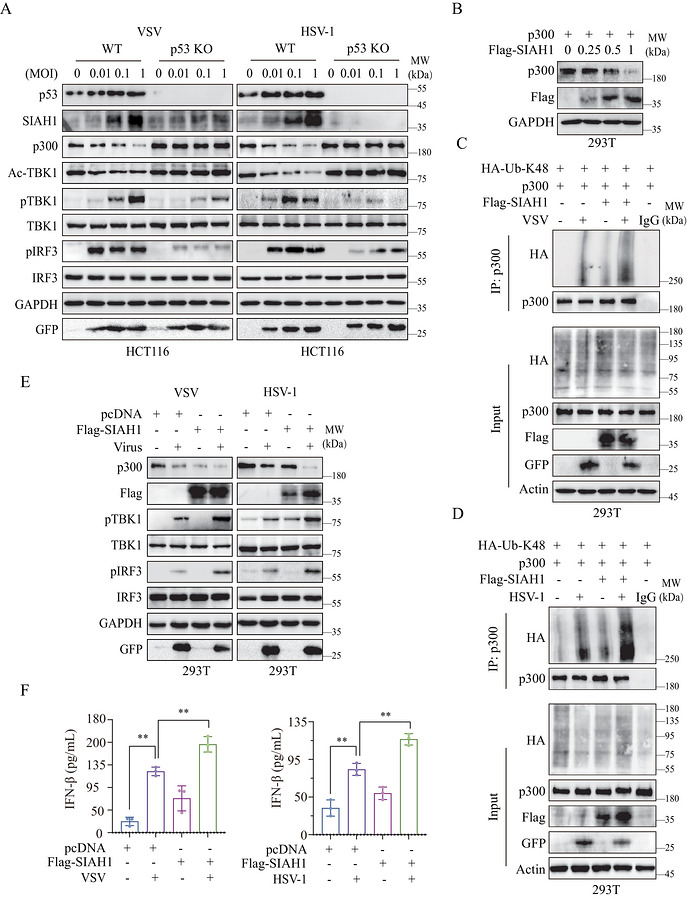
Virus infection activates the p53‐SIAH1 axis to degrade p300. (A) p53 enhances TBK1 and IRF3 phosphorylation. Wild‐type (WT) and p53‐deficient (p53 KO) HCT116 cells were infected with the indicated MOIs of VSV or HSV‐1 and incubated for 12 h. Cell lysates were prepared and analyzed by Western blot for TBK1 and IRF3 phosphorylation and TBK1^K241^ acetylation and for p300, p53, and SIAH1 expression. (B) SIAH1 downregulates p300 expression. 293T cells were co‐transfected with the p300 expression vector and increasing amounts of Flag‐tagged SIAH1 (Flag‐SIAH1). The pcDNA3.1 empty vector was supplemented to ensure equal amounts of total DNA in each transfection. After incubation for 48 h, cell lysates were prepared and analyzed for p300 and SIAH1 expression by Western blot with their specific antibodies. (C, D) SIAH1 enhances p300 K48 polyubiquitination. 293T cells were co‐transfected with the p300 and HA‐Ub‐K48 vectors plus the empty vector or the SIAH1 expression vector. After incubation for 36 h, the cells were left uninfected or infected with VSV or HSV‐1 (1 MOI each). After incubation for 12 h in the presence of MG132 (10 µm), cell lysates were immunoprecipitated with an anti‐p300 antibody and analyzed for p300 ubiquitination by Western blot with an anti‐HA antibody. Whole cell lysates were analyzed for the expression of indicated proteins by Western blot as input controls. (E) SIAH1 enhances TBK1 and IRF3 phosphorylation. 293T cells were transfected with an empty vector or the Flag‐SIAH1 expression vector. After incubation for 36 h, the cells were left uninfected or infected with VSV or HSV‐1 (1 MOI each). After incubation for 12 h, cell lysates were prepared and analyzed for the indicated proteins by Western blot with their specific antibodies. (A–E) Representative blots from one of three independent experiments with similar results are shown. (F) SIAH1 enhances IFN production. 293T cells were transfected with an empty vector or the Flag‐SIAH1 plasmid and incubated for 24 h. The cells were then left uninfected or infected with VSV or HSV‐1 (1 MOI each). After incubation for 12 h, conditioned media were collected and analyzed by IFN‐β ELISA. Data represent the mean ± SD from three independent experiments. An unpaired Student's *t*‐test was used to determine the statistical significance. ^**^ *p* < 0.01.

SIAH1 is a p53‐inducible E3 ubiquitin ligase that has previously been shown to target p300 [33]. We next tested if virus infection and immune activation may downregulate p300 expression by inducing p53 and SIAH1 expression. HCT116 cells are a human colon cancer cell line that harbors the functional wild‐type *p53* gene. The *p53* gene is completely knocked out in this cell line, as reported in our prior publication [32]. VSV and HSV‐1 infection dose‐dependently induced p53 and SIAH1 expression but downregulated p300 expression and TBK1 acetylation in parental HCT116 cells (Figure 7A). In contrast, VSV and HSV‐1 induced little or no SIAH1 expression at all and failed to downregulate p300 and TBK1 acetylation in p53‐deficient HCT116 cells (Figure 7A). p53 knockout also suppressed TBK1 and IRF3 phosphorylation in HCT116 cells infected with VSV or HSV‐1 (Figure 7A). These observations suggest that virus infection downregulates p300 expression by inducing p53 and SIAH1.

The role of SIAH1 in downregulating p300 expression was confirmed by the observation that SIAH1 transfection dose‐dependently decreased the levels of p300 in HCT116 cells co‐transfected with the p300 expression vector (Figure 7B). Virus infection and SIAH1 overexpression alone enhanced p300 K48 polyubiquitination, whereas virus infection in combination with SIAH1 transfection further increased p300 K48 polyubiquitination (Figure 7C,D). HSV‐1 infection did not increase p300 polyubiquitination at the linkage of K6, K11, K29, K33, K63 (Figure S6). SIAH1 overexpression enhanced VSV‐ or HSV‐1‐induced TBK1 and IRF3 phosphorylation (Figure 7E) and increased the levels of IFN‐β (Figure 7F). These observations collectively suggest that the p53‐SIAH1 axis plays an important role in downregulating p300 expression and enhancing innate antiviral immunity.

## 3 Discussion

Our present study focuses on the mechanisms of regulation of innate immunity by p300 and p53. We provide evidence that p300 suppressed antiviral responses in vitro and in vivo by acetylating TBK1 and inhibiting its activation. In contrast, p53 enhanced antiviral responses by inducing SIAH1 expression and subsequently targeting p300 for polyubiquitination and proteasomal degradation. Our study provides mechanistic insights into how a well‐characterized histone acetyltransferase and a tumor suppressor are integrated into a common pathway to regulate innate antiviral immunity.

TBK1 activation is regulated by a variety of post‐translational modifications including ubiquitination, acetylation, and phosphorylation [34, 35, 36]. TBK1 is acetylated at K241 and K692 [11, 12]. TBK1 acetylation leads to poor TBK1 phosphorylation and activation [11, 12]. Gcn5 and PCAF, two redundant histone acetyltransferases that interact with TBK1, are not responsible for acetylating TBK1 [37]. Our study provides several lines of evidence that p300 is the enzyme that acetylates TBK1: (1) A‐485 and p300 knockdown inhibited TBK1 acetylation, whereas p300 overexpression enhanced TBK1 acetylation; (2) Lysine substitution at position 241 and/or 692 of TBK1 abrogated p300‐mediated acetylation; (3) The N‐terminal region of p300 bound TBK1. The IBiD domain of p300 that binds IRF3 is located in the C‐terminus. This suggests that p300 directly interacts with TBK1, and that p300 acetylates TBK1 independent of IRF3.

p300 interacts with various transcription factors and acetylates histones in the transcription factor binding region [14]. Newcastle virus (NDV) infection induces IRF3 association with p300 and recruits it into the IFN‐β promoter, where it hyperacetylates histones H3 and H4 [16, 17]. A dominant‐negative IRF3, which does not bind p300, inhibits SeV‐induced IFN‐β transcription [17]. The large yellow croaker IRF11 (LcIRF11) recruits p300 and RNA polymerase II to the MDA5 promoter to increase histone H3K27 acetylation and MDA5 gene transcription [38]. Later studies showed that several viral proteins can disrupt the p300‐IRF3 interaction to suppress IFN‐β gene expression. For example, the D129L protein of African swine fever virus binds CBP/p300 to inhibit its interaction with IRF3 and IFN‐β gene expression [39]. Propionylated viral interferon regulatory factor 1 (vIRF1) encoded by Kaposi's sarcoma‐associated herpesvirus (KSHV) disrupts the IRF3‐p300/CBP interaction and inhibits the type I IFN response [40]. The Epstein‐Barr virus nuclear antigen 3A (EBNA3A) interacts with p300 to limit the binding of IRF3 to the IFN‐β promoter, thereby hampering downstream type I IFN signaling [41]. While these observations suggest that p300 positively regulates innate antiviral immunity, there is no direct evidence, such as using p300‐deficient cells, to prove that p300 is indeed required for IFN‐β gene transcription. Our present study showed that p300 acetylated TBK1 and inhibited its activation; pharmacological inhibition and genetic ablation of p300 enhanced IFN‐β gene transcription and inhibited virus replication, whereas p300 overexpression suppressed innate antiviral responses. We conclude that p300 overall functions as a negative regulator of innate immunity. In line with this supposition, Zeng et al. [18] recently reported that p300 compromises antitumor immunity by increasing SOCS1 expression and inhibiting STAT1 activation; p300‐deficiency enhances IFN‐β and MHC‐I expression and antigen presentation.

**FIGURE 8:**
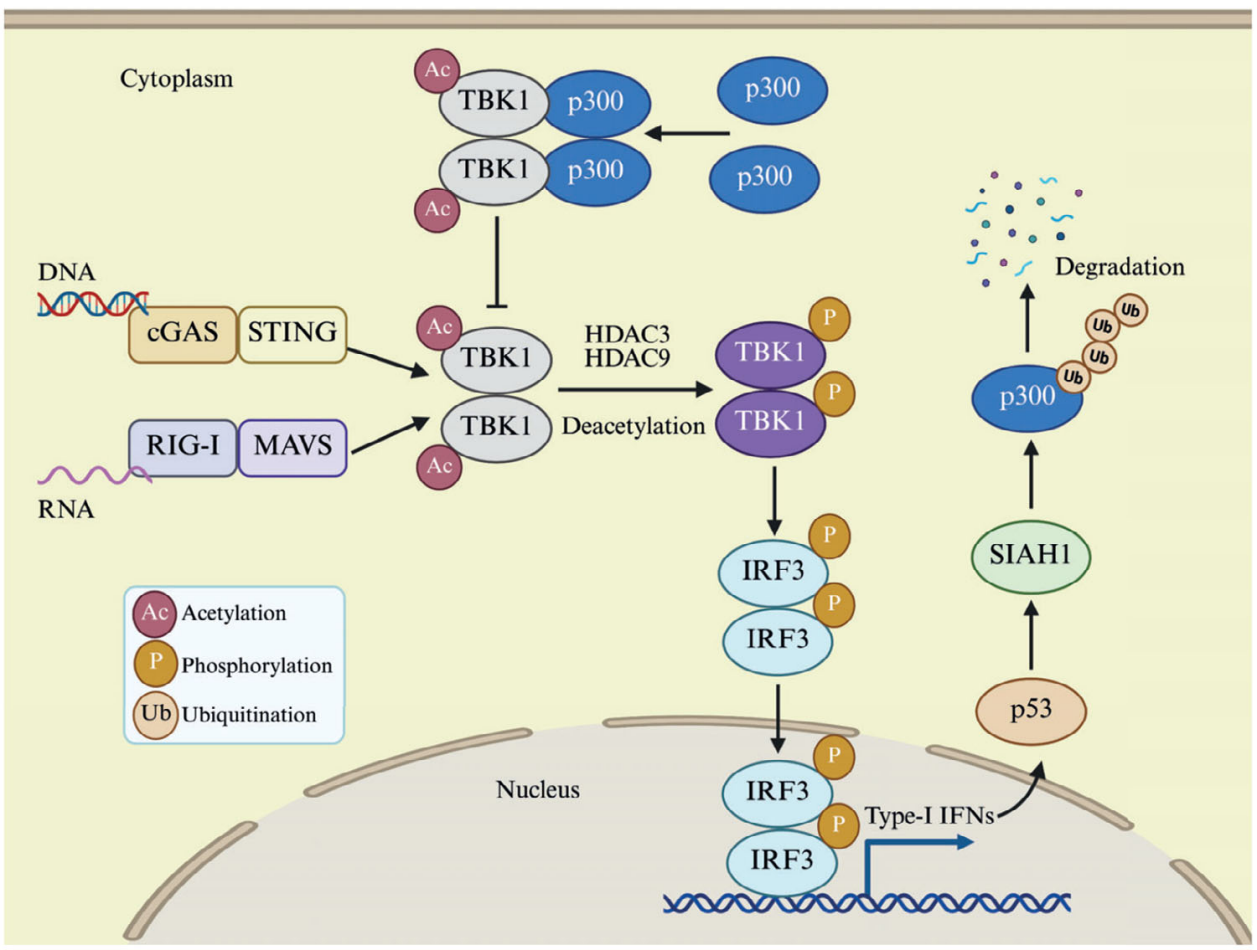
Schematic illustration of p300‐mediated suppression of antiviral immunity. During the resting stage, the inactive form of TBK1 is present as a homodimer in the cytoplasm. TBK1 interaction with p300 is likely to induce p300 dimerization and activation. Activated p300 acetylates TBK1 and inhibits its activation. Upon sensing cytosolic nucleic acid, some TBK1 dimers, which are deacetylated by HDAC3 and HDAC9, become activated by the cGAS‐STING pathway or the RIG‐I‐MAVS pathway. TBK1 phosphorylates and activates IRF3 to induce IFN gene expression. Type I interferons induce p53 expression. p53 functions as a transcription factor to induce the expression of the SIAH1 ubiquitin ligase. SIAH1 induces p300 K48‐linked polyubiquitination and proteasomal degradation. p300 downregulation leads to decreased TBK1 acetylation but increases TBK1 activation. The p53‐SIAH1 axis sustains immune activation by downregulating p300 expression.

SIAH1 is an E3 ubiquitin ligase that has been implicated in suppressing virus replication by various mechanisms. For example, SIAH1 induced by p53 through all‐trans retinoic acid (ATRA) or reactive oxygen species (ROS) targets hepatitis B virus (HBV) X protein (HBx) to inhibit HBV replication [42, 43, 44]. SIAH1 inhibits HIV replication by decreasing ELL2 expression and HIV gene transcription [45, 46]. SIAH1 inhibits rice reovirus replication by targeting the capsid protein [47]. SIAH1 targets the p10 subunit of avian reovirus to inhibit viral replication [48]. SIAH1 inhibits influenza virus (PR8 strain) replication by targeting USP19 to enhance MAVS, TRAF3, and TRIF K63 de‐ubiquitination and innate immunity [27]. Our present study shows that SIAH1 expression was induced by viral infection in a p53‐dependent manner, and that SIAH1 was responsible for p300 K48 polyubiquitination. Since p300 was implicated in acetylating TBK1 and inhibiting its activation, we propose that SIAH1 may inhibit VSV and other virus replication in part by inducing p300 degradation and subsequently enhancing innate immunity (Figure 8).

In addition to being regulated by SIAH1‐mediated K48‐linked ubiquitination, p300 stability is also regulated by some deubiquitinating enzymes. For example, several ubiquitin‐specific peptidases (USPs) including USP12, USP24, and UCHL3 are capable of removing K48 polyubiquitin and increasing p300 stability [49, 50, 51]. We conducted preliminary experiments to investigate their role in regulating p300 expression. We found that overexpression of UCHL3 but not USP12 and USP24 prevented VSV infection‐induced p300 degradation (Figure S7A). VSV infection did not decrease or only weakly decreased the levels of USP12, USP24, and UCHL3 mRNA (Figure S7B). Western blot analysis revealed that VSV infection indeed weakly decreased UCHL3 protein expression (Figure S7C). These observations suggest that virus infection may regulate p300 stability in part by decreasing UCHL3 expression.

p53 is best known for its tumor suppressor function [19, 20]. However, p53 also contributes to the regulation of antiviral and antitumor immunity [21]. p53 directly activates the expression of immune response genes such as IRF9, CCL2, PKR, and ISG15. p53‐deficient mice are more permissive to Sendai and influenza virus infections and produce 100‐fold less IFN than wild‐type mice [23]. p53 is involved in a positive feedback loop in which p53 enhances antiviral immunity, whereas IFN‐αβ and IFN‐λ induce p53 expression [21, 52]. Our present study shows that VSV and HSV‐1 infection induced p53 and SIAH1 expression, p53 knockout prevented virus infection‐induced SIAH1 expression, and SIAH1 overexpression induced p300 ubiquitination and proteasomal degradation. Since p300 acetylated TBK1 and inhibited its activation, we propose that p53 enhances innate antiviral immunity in part by the p53‐SIAH1‐p300‐TBK1 pathway (Figure 8).

We are aware of a couple of weaknesses in our present investigation. First, we did not dissect the relative contribution of p300‐acetylated TBK1 and histones to IFN gene transcription. p300 may play a dual but opposing role in regulating IFN production: it inhibits TBK1 and IRF3 activation but enhances IFN gene transcription initiation by opening chromatin accessibility in the IFN promoter regions. Second, we did not investigate whether CBP, a paralog of p300 that shares an overall 85% amino acid identity in its catalytic region with p300 [13, 14], plays a redundant role in acetylating TBK1. Notably, CBP acetylates HMG I(Y) and destabilizes the enhanceosome assembly, thus turning off IFN‐β gene expression [53]. CBP acetylates STAT1 and inhibits its transcriptional activity [54]. Whether CBP suppresses innate immunity in part by acetylating TBK1 remains to be investigated.

## 4 Conclusions

p300 has been long recognized as a histone acetyltransferase that positively regulates IFN responses. Contrary to the established paradigm, our present study provides compelling evidence that p300 negatively regulates innate antiviral immunity. Mechanistically, p300 acetylates TBK1 at K241 and K692 to inhibit its phosphorylation and activation. Moreover, p300‐mediated suppression of innate antiviral immunity is alleviated by the p53‐SIAH1 axis, which downregulates p300 expression through proteasomal degradation. Our study reveals a previously unrecognized mechanism of regulation of the IFN response by p300 and p53.

## 5 Materials and Methods

### 5.1 Reagents and Antibodies

A‐485 was purchased from Selleck Chemicals (Houston, Texas, USA). Poly (dA:dT) (tlrl‐patn) and poly (I:C) (tlrl‐pic) were purchased from InvivoGen (San Diego, California, USA). The Cell Counting Kit‐8 (CCK‐8) was purchased from Beyotime Biotechnology (Shanghai, China). TurboFect Transfection Reagent, FastDigest *Nhe*I and *Bam*HI restriction enzymes were purchased from Thermo Scientific (Waltham, Massachusetts, USA). pEASY‐Uni Seamless Cloning and Assembly Kit was purchased from Transgen (Beijing, China). Antibodies for phospho‐TBK1 (Ser172) (#5483), TBK1 (#3504), phospho‐IRF3 (Ser396) (4D4G) (#4947), IRF3 (D83B9) (#4302), p300 (D8Z4E) (#86377), HA‐tag (6E2) (#2367), HRP‐conjugated anti‐Rabbit IgG (#7074S), and HRP‐conjugated anti‐mouse IgG (#7076S) were purchased from Cell Signaling Technology (Danvers, Massachusetts, USA). A horseradish peroxidase (HRP)‐conjugated anti‐rabbit IgG (#002‐05) was purchased from Jackson ImmunoResearch Laboratories (West Grove, Pennsylvania, USA). An anti‐SIAH1 antibody (ab2237) was purchased from Abcam (Cambridge, UK). An anti‐UCHL3 antibody (12384‐1‐AP) was purchased from Proteintech (Wuhan, China). An anti‐p53 (Ab‐3) antibody (OP29) was purchased from Calbiochem (Merck Millipore/Sigma–Aldrich). An anti‐green fluorescence protein (GFP) mouse monoclonal antibody (HT801) and an anti‐Flag mouse monoclonal antibody (HT201) were purchased from TransGen Biotech (Beijing, China). An anti‐GAPDH antibody (sc‐47724) was purchased from Santa Cruz Biotechnology, Inc. (Santa Cruz, California, USA). An anti–acetyl‐lysine antibody was purchased from Proteintech (Wuhan, China). The anti‐acetylated TBK1 (K241) antibody was kindly provided by Professor Qian Zhang (Naval Medical University, Shanghai, China).

### 5.2 Cell Culture and Viruses

L929 (RRID: CVCL_0462), 293T (RRID: CVCL_0063), HT1080 (RRID: CVCL_0317), NL20 (RRID: CVCL_3756), RAW264.7 (RRID: CVCL_0493), and Vero (RRID: CVCL_0059) cell lines were obtained from the American Type Culture Collection (ATCC, Manassas, Virginia, USA). L929, HT1080, RAW264.7, and 293T cells were cultured in DMEM supplemented with 10% fetal bovine serum (FBS). NL20 cells were grown in Ham's F‐12 medium supplemented with 1.5 g/L sodium bicarbonate, 2.7 g/L glucose, 2.0 mm L‐glutamine, 0.1 mm nonessential amino acids, 5 µg/mL insulin, 10 ng/mL epidermal growth factor, 1 µg/mL transferrin, 500 ng/mL hydrocortisone, and 4% FBS. Vero cells were cultured in α‐MEM supplemented with 10% FBS at 37°C in a humidified incubator with 5% CO_2_. HCT116 cells were kindly provided by the Cell Bank of the Chinese Academy of Sciences. p53^−/−^ HCT116 cells were a gift from Dr. Bert Vogelstein (Johns Hopkins University, Maryland, USA). HCT116 and p53^−/−^ HCT116 cells were cultured in McCoy's 5a medium supplemented with 10% fetal bovine serum. SeV, VSV, and HSV‐1, which all express a green fluorescent protein (GFP) fusion protein, were kindly provided by Professor Jianzhong Zhu (Yangzhou University, Yangzhou, China). VSV used for animal experiments was prepared and reported in our prior publication [55]. Murine primary alveolar epithelial cells were isolated by mincing lung tissues into approximately 0.5 mm^3^ fragments, followed by three washes with Hank's balanced salt solution (HBSS). The tissue fragments were then digested in HBSS containing trypsin (10 000 U/mL), elastase (5.0 U/mL), and DNase I (10 000 U/mL) at 37°C for 45 min with gentle agitation. The resulting cell suspension was filtered through a 70‐µm nylon mesh and subjected to three rounds of differential adherence to deplete adherent macrophages and fibroblasts. The purified epithelial cells were cultured in DMEM/F12 medium supplemented with hydrocortisone (500 ng/mL), transferrin (1 mg/mL), insulin (5 µg/mL), EGF (10 ng/mL), and 0.1 mm non‐essential amino acids.

### 5.3 Plasmids

The pCMVβ‐p300‐Myc expression vector (#30489) was obtained from Addgene (Watertown, Massachusetts, USA). The N‐terminal (p300‐N), core (p300‐Core), and C‐terminal (p300‐C) fragments of p300 were amplified by PCR using the Myc‐p300 plasmid as the template. cDNA templates reverse‐transcribed from total RNA extracted from 293T cells were used to amplify SIAH1, USP12, USP24, and UCHL3. The primers used for PCR amplification are listed in Table S1. PCR products were purified and inserted into the *Nhe*I (FD0973, Thermo Scientific) and *Bam*HI (FD0055, Thermo Scientific) sites of the pcDNA3.1‐Flag vector via homologous recombination (CU201‐02, TransGen). Site‐directed mutagenesis of TBK1 was performed using the Fast Mutagenesis Kit (FM111‐01, TransGen). The primers used for site‐directed mutagenesis are listed in Table S1. For the Bimolecular Fluorescence Complementation (BiFC) assay, the TBK1 gene was cloned into the N159 expression vector, and the p300 gene was cloned into the C160 expression vector using *EcoR*I (FD0274, Thermo Scientific) and *Bam*HI (FD0055, Thermo Scientific) restriction enzymes. The HA‐TBK1 expression vector was kindly provided by Dr. Shilei Zhang (Lanzhou Veterinary Research Institute, Chinese Academy of Agricultural Sciences, Lanzhou, China). The HA‐Ub‐K6, HA‐Ub‐K11, HA‐Ub‐K27, HA‐Ub‐K29, and HA‐Ub‐K33 plasmids were kindly provided by Prof. Daxin Peng (Yangzhou University, Yangzhou, China). The HA‐Ub‐WT, HA‐Ub‐K63, HA‐Ub‐K48, and the luciferase reporter plasmids driven by the IFN‐β promoter and IRF3 binding sites were generously provided by Prof. Jianzhong Zhu (Yangzhou University, Yangzhou, China).

### 5.4 Western Blot

Cells cultured in 12‐well plates were harvested and lysed in NP‐40 lysis buffer (50 mm Tris‐HCl, pH 8.0; 150 mm NaCl; 1% NP‐40; 5 mm EDTA; 10 µg/mL aprotinin; 10 µg/mL leupeptin; and 1 mm phenylmethylsulfonyl fluoride). After sonication, cell lysates were centrifuged at 15 000 rpm for 15 min. The supernatants were collected and analyzed for the proteins of interest by Western blotting with specific antibodies, followed by incubation with horseradish peroxidase‐conjugated goat anti‐rabbit or anti‐mouse IgG and SuperSignal Western Pico enhanced chemiluminescence substrate (Pierce Chemical Co., Rockford, Illinois, USA). All Western blot experiments were repeated at least twice, each with the detection of β‐actin or GAPDH as a loading control. Protein phosphorylation was first analyzed by probing with an antibody against phosphorylated protein. The membrane was stripped and re‐probed with an antibody against the total protein. The relative phosphorylation levels were analyzed by quantifying the density of the phosphorylated protein bands normalized to their corresponding total proteins. The results are presented as bar graphs.

### 5.5 IFN‐β ELISA

L929 cells were incubated in the absence or presence of A‐485 (5 µm) for 6 h and were then left uninfected or infected with VSV or HSV‐1 (1 MOI each) for 12 h, or transfected with poly (I:C) (1 µg) or poly (dA:dT) (1 µg) for 8 h. Conditioned media were collected, and IFN‐β levels were quantified by ELISA kit (#E‐EL‐M0033, Elabscience) (Wuhan, China). Data are presented as mean ± standard deviation (SD) from three independent experiments. IFN‐β production in RAW264.7 and NL20 cells was similarly analyzed as above except with 10 µm A‐485.

### 5.6 Cytotoxicity Assay

L929 cells were seeded in 96‐well plates and cultured in medium containing the indicated concentrations of A‐485 (100 µL per well) in triplicate for 48 h. CCK‐8 reagent (C0037, Beyotime) was then added, followed by incubation for an additional 4 h. Absorbance at 450 nm was measured using a microplate reader.

### 5.7 Virus Titration

The samples of conditioned media and tissue lysates were diluted in a 10‐fold series (10^1^–10^9^) and used to infect Vero cells. The 50% tissue culture infection dose (TCID_50_/100 µL) values were determined by using the standard Reed and Muench method.

### 5.8 CRISPR/Cas9 Silencing

p300 silencing was performed by transfecting L929 cells with a CRISPR/Cas9 expression vector encoding a single guide RNA (sgRNA) targeting the mouse p300 gene. The targeting sequence was as follows: mP300, 5’‐CTCTCGGCGTCCGCCAGCGA‐3’. Complementary oligonucleotides containing 4 extra nucleotides at each end were annealed and ligated into the *Bsm*BI‐digested LentiCRISPRv2 vector using T4 DNA ligase. L929 cells were seeded in a 24‐well plate and transfected with the LentiCRISPRv2 vector or the vector encoding p300 sgRNA using TurboFect transfection reagent according to the manufacturer's instructions. After 6 h of incubation, the transfection medium was replaced with fresh complete medium. Forty hours later, cells were harvested, reseeded in 6‐well plates, and selected with puromycin (3 µg/mL). Fresh puromycin‐containing medium was replaced every 3 days. After 3 weeks of selection, individual clones were isolated, expanded, and analyzed for p300 expression by Western blot. At least two clones confirmed to lack p300 expression were used for subsequent experiments to investigate the role of p300 in innate immune signaling.

### 5.9 Co‐Immunoprecipitation

Cells were lysed in 1% Triton X‐100 lysis buffer (20 mm Tris‐HCl, pH 7.5; 100 mm NaCl; 1% Triton X‐100; 10% glycerol; 10 mm EDTA) supplemented with protease and phosphatase inhibitor cocktail (P002, NCM Biotech) on ice, followed by sonication. After centrifugation at 12 000 ×g for 15 min at 4°C, the supernatant was collected. For immunoprecipitation, rabbit anti‐TBK1, anti‐p300, or mouse anti‐Flag antibodies were first incubated with BeyoMag Protein A+G magnetic beads (P2108, Biyuntian, China) at 4°C for 2 h with rotation to allow antibody binding to the beads. For the negative control, species‐matched normal rabbit or mouse IgG was used under the same conditions. The antibody‐bound beads were then incubated with cell lysates overnight at 4°C with gentle rotation. Immune complexes were washed three times with lysis buffer using a magnetic stand and subsequently boiled in 2× loading buffer for 5 min. Co‐immunoprecipitated proteins were analyzed by Western blot using specific antibodies against p300, TBK1, or Flag, respectively.

### 5.10 Bimolecular Fluorescence Complementation (BiFC) Assay

The BiFC assay was conducted according to the published protocol [56]. Briefly, 293T cells seeded on coverslips in 24‐well plates were co‐transfected with the C160‐p300 vector plus the N159‐TBK1 vector. The C160 and N159 empty vectors were used as a negative control, whereas the C160‐p53 and N159‐SV40gp6 vectors were used as a positive control [32]. After incubation for 48 h, the cells were fixed with ice‐cold methanol for 15 min. After rinsing three times in PBS, the cells were blocked with 5% goat serum for 1 h at room temperature. Nuclei were counterstained with DAPI (10 µg/mL) in the dark for 10 min, followed by three washes with PBS. Coverslips were mounted with antifade mounting medium, sealed with nail polish, and examined under a confocal laser microscope. The BiFC signal was quantified as the ratio of the red fluorescence area to the total cell area using ImageJ (NIH). Data represent the mean ± SD of three randomly selected fields from one representative experiment of three independent experiments.

### 5.11 Real‐Time Quantitative PCR Analysis

Total RNA was extracted from L929 cells and from tissues using TRIzol reagent (Invitrogen). RNA integrity was verified by agarose gel electrophoresis. Reverse transcription was performed using the HiScript III RT SuperMix for qPCR (R323, Vazyme) following the manufacturer's instructions. Quantitative real‐time PCR reactions with primers (Table S2) were carried out with 2× ChamQ Universal SYBR qPCR Master Mix (Q711, Vazyme). β‐Actin was amplified as an internal control. Relative mRNA expression levels were calculated using the 2−ΔΔCT method, normalized to β‐actin expression. Fold changes were determined by comparing normalized values of treated samples with those of untreated controls. All qRT‐PCR reactions were performed in triplicate. Data from three independent experiments or from four mice were pooled for statistical analysis.

### 5.12 Luciferase Reporter Assay

293T cells seeded in 96‐well plates were transfected with a luciferase reporter driven by multiple IRF3 binding sites or by the IFN‐β promoters. A β‐actin promoter‐driven *Renilla* luciferase plasmid was co‐transfected as an internal control. After incubation for 24 h, the cells were pre‐treated with A‐485 (5 µm) and incubated for 6 h. Cells were then infected with VSV or HSV‐1 (1 MOI each) and incubated for an additional 12 h. Luciferase activity was assayed using the Dual‐Luciferase Reporter Assay Kit (TransDetect, Beijing, China) and quantified with a Tecan plate reader (Phenix Research Products, Phenix, AZ). Firefly luciferase activity was normalized to Renilla luciferase activity. The results were presented as means ± SD from triplicate wells. All experiments were independently repeated at least twice with similar results. To further determine the inhibitory effect of p300 in regulating IFN gene expression, 293T cells were transfected with pcDNA3.1 or the p300 expression vector plus a luciferase reporter driven by multiple IRF3 binding sites or by the IFN‐β promoters. A β‐actin promoter‐driven *Renilla* luciferase plasmid was co‐transfected as an internal control. After incubation for 36 h, the cells were left uninfected or infected with SeV or VSV (1 MOI each) and then incubated for 12 h. Luciferase activity was analyzed as above.

### 5.13 Animals

All animal experiments were approved by the Institutional Animal Care and Use Committee of Yangzhou University (Approval number 202403064; date of approval: March 1, 2024) and conducted in accordance with the Guide for the Care and Use of Laboratory Animals by the National Research Council. Heterozygous p300^flox/+^ and Sftpc‐CreER^T2+/−^ mice were purchased from Cyagen Biosciences (Suzhou, China). Mice were bred to ultimately obtain p300^flox/flox^ mice carrying the Sftpc‐CreER^T2^ (p300^flox/flox^ Sftpc‐CreER^T2^). Male p300^flox/flox^Sftpc‐CreER^T2^ mice at 6 weeks of age were used for all experiments. Conditional knockout was performed according to a prior study [51]. Briefly, Tamoxifen (Tam) (MCE, ICI 47699, Shanghai, China) was dissolved in corn oil (20 mg/mL) and administered intraperitoneally at a dose of 120 mg/kg body weight once daily for 7 consecutive days to induce gene deletion. Mice in the control group received an equal volume of corn oil alone. After a 7‐day resting period post‐treatment, mice were infected with VSV via intraperitoneal injection at a dose of 2 × 10^8^ plaque‐forming units (PFU)/kg body weight. Mice were euthanized 16 h post‐infection by cervical dislocation. Lung, liver, and spleen tissues were collected for further analysis. The sections (5 µm) of lung tissue blocks were stained with hematoxylin and eosin (H & E). The inflammatory areas in each section of whole lungs were quantified by using CaseViewer 2.0 software (3DHistech, Budapest, Hungary). For survival analysis, mice were treated with Tamoxifen or vehicle as described above. One week later, mice were intraperitoneal infected with VSV at a dose of 2 × 10^9^ PFU/kg body weight under anesthesia. Body weight was monitored daily, and mice were considered moribund and euthanized when body weight dropped below 80% of the initial weight. For HSV‐1 infection, tamoxifen‐ or vehicle‐treated mice were intranasally infected with HSV‐1 at a dose of 2 × 10^6^ PFU/mouse under anesthesia. Mice were euthanized 24 h post‐infection by cervical dislocation. Lung tissues were collected and analyzed for antiviral responses.

### 5.14 Statistical Analysis

Differences in blot density, mRNA levels, luciferase activity, IFN‐β, BiFC quantitative data, GFP fluorescence intensity, and virus titers were analyzed using the unpaired two‐tailed Student's *t*‐test. Data are presented as mean ± SD unless otherwise indicated. Differences in survival were analyzed using the Log‐Rank test. The sample size and the specific statistical test used are indicated in the corresponding figure legends. Statistical significance was defined as ^*^ *p* < 0.05, ^**^ *p* < 0.01, and ns, not significant. All analyses were performed using GraphPad Prism 8.0.2. (GraphPad Software; https://www.graphpad.com/scientific‐software/prism).

## Author Contributions

H.Y.: Designed and performed experiments, interpreted data, conducted statistical analysis, drafted the manuscript. Z.Z.: Designed and performed experiments, collected and analyzed data. X.P.: Conducted experiments. X.Z.: Provided resources and experimental advice. P.L.: Performed supervision and project administration. J.S.: Performed experimental coordination and project administration. X.X.: Initiated and conceived the study, performed supervision and execution of the project, grant procurement, manuscript writing and editing. All authors have read and approved the manuscript.

## Funding

This research was supported in part by the National Natural Science Foundation (32573372), Paragon Philanthropy, and the Priority Academic Program Development of Jiangsu Higher Education Institutions to Xiulong Xu, and the Postgraduate Research & Practice Innovation Program of Jiangsu Province (Yangzhou University) (KYCX22‐3547) to Huidi Yu.

## Ethics Statement

All animal experiments were approved by the Institutional Animal Care and Use Committee of Yangzhou University (Approval number 202403064; date of approval: March 1, 2024) and conducted in accordance with the Guide for the Care and Use of Laboratory Animals by the National Research Council.

## Conflicts of Interest

All authors declare no conflicts of interest.

## Supporting information




**Supporting File 1**: advs76101‐sup‐0001‐SuppMat.pdf.

**Supporting File 2**: advs76101‐sup‐0002‐TablesS1‐S2.docx.

## Data Availability

The data that support the findings of this study are available from the corresponding author upon reasonable request.
