# Supplementary material for: p300 Degradation by the p53‐SIAH1 Axis Relieves TBK1 Acetylation to Enhance Innate Antiviral Immunity
Source: Adv Sci (Weinh). 2026 Jun 15;13(50):e76101. doi: 10.1002/advs.76101 (PMC13336735; doi:10.1002/advs.76101)
Supplement: Supplementary file 1 [file ADVS-13-e76101-s002.pdf]

Figure S1

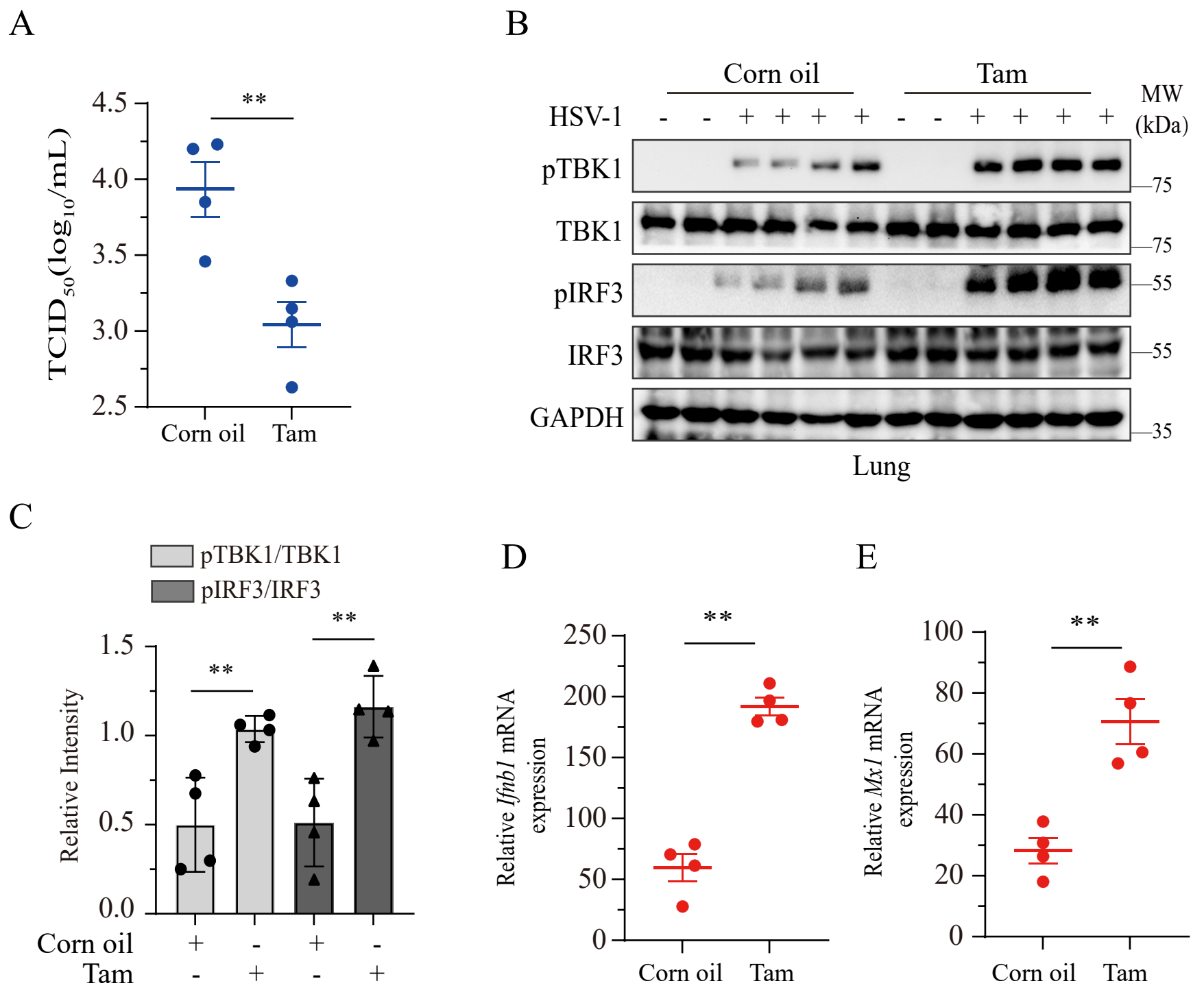

Figure S1. p300 deficiency enhances antiviral responses against HSV-1 infection in vivo. **(A)** p300 deficiency inhibits HSV-1 replication. Control and p300 cKO mice (male, 6-7 weeks old) were left uninfected (2 mice per group) or intranasally infected with HSV-1 at a dose of 2 × 10^6 PFU/mouse (4 mice per group). Mice were sacrificed at 24 h post-infection, and lung tissues were collected for viral titer analysis by $TCID_{50}$ assay. **(B & C)** p300 deficiency enhances HSV-1-induced TBK1 and IRF3 phosphorylation. Lung tissue lysates were analyzed for TBK1 and IRF3 phosphorylation by Western blot **(B)**. Relative TBK1 and IRF3 phosphorylation levels were quantified using ImageJ and normalized to their corresponding total proteins **(C)**. **(D & E)** p300 deficiency enhances HSV-1-induced antiviral gene transcription. Total RNA was extracted from lung tissues, and the mRNA levels of *Ifnb1* and *Mx1* were analyzed by RT-PCR. Data in (A, C, D, E) are presented as mean ± SD from 4 mice per group. An unpaired Student's *t*-test was used to determine the statistical significance. $**p < 0.01$.

Figure S2

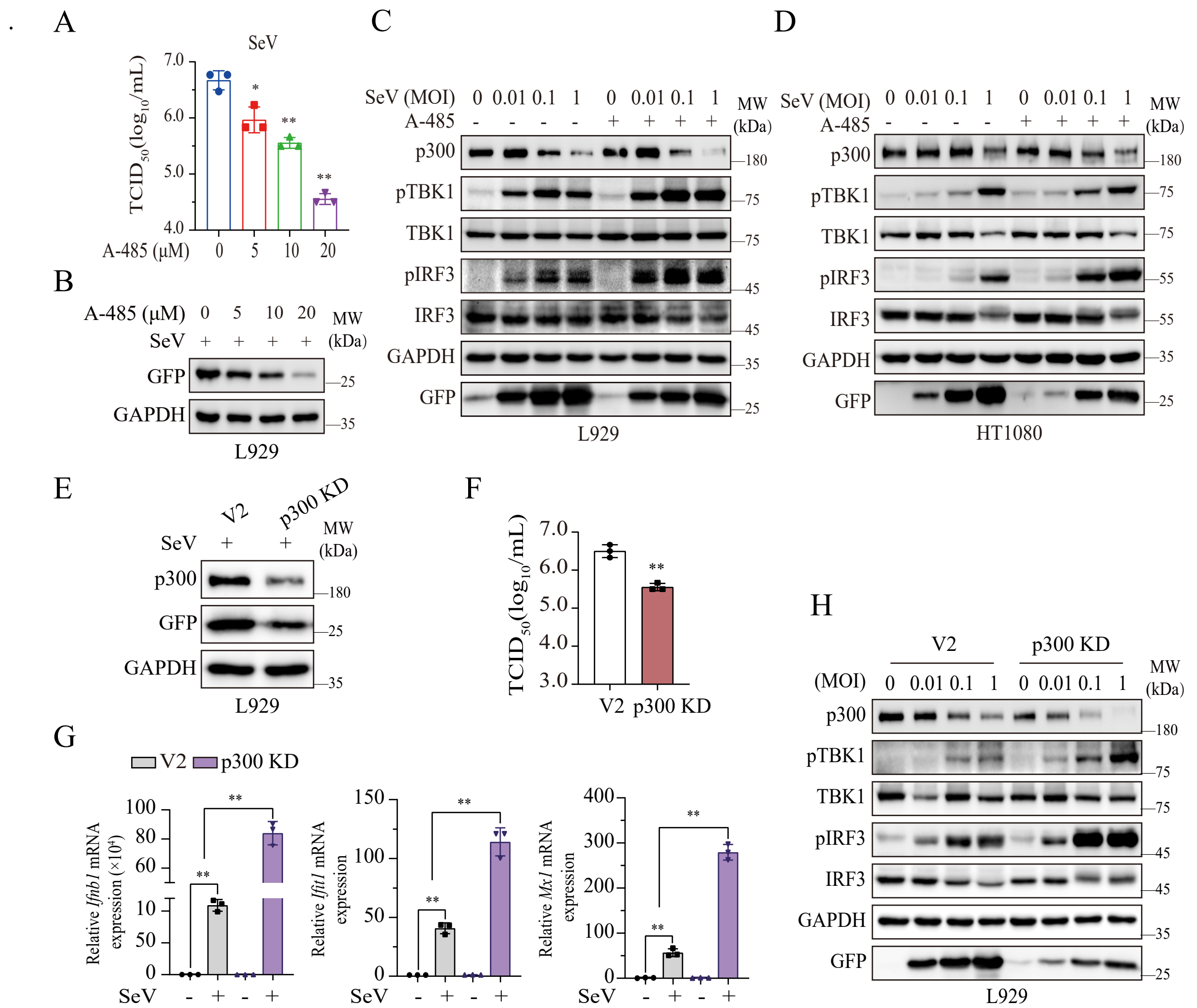

Figure S2. p300 inhibition promotes antiviral immunity and attenuates SeV replication. (**A & B**) A-485 inhibits SeV replication. L929 cells pre-treated with the indicated concentrations of A-485 (0, 5, 10, and 20 μM) for 16 h were infected with SeV (0.02 MOI). After incubation for 16 h, conditioned media were collected and analyzed for viral titers by $TCID_{50}$ assay (**A**). Data represent mean ± SD from three independent experiments. An unpaired Student's *t*-test was used to determine the statistical significance. $^{**}p < 0.01$. Cell lysates were prepared and analyzed for GFP-tagged viral protein expression by Western blot using an anti-GFP antibody (**B**). (**C & D**) A-485 enhances SeV-induced TBK1 and IRF3 phosphorylation. L929 and HT1080 cells were incubated in the absence or presence of A-485 (5 μM) for 6 h and then left uninfected or infected with the indicated MOIs of SeV for 12 h. Cell lysates were prepared and analyzed for the indicated proteins by Western blot. (**C-D**) Representative blots from one of three independent experiments with similar results are shown. **(E & F)** p300 knockdown inhibits SeV replication. Control (V2) and p300 knockdown (p300 KD) L929 cells were infected with SeV (0.02 MOI). After incubation for 16 h, conditioned media were collected and analyzed for viral titers by $TCID_{50}$ assay. Data represent mean ± SD from three independent experiments. An unpaired Student's *t*-test was used to determine the statistical significance. $^{**}p < 0.01$. Cell lysates were analyzed for p300 and GFP expression by Western blot (**F**). Representative blots from one of three independent experiments with similar results are shown. **(G)** p300 knockdown enhances SeV-induced IFN-β and ISG mRNA expression. Control (V2) and p300 KD L929 cells were infected with 1 MOI of SeV and incubated for 12 h. The levels of *Ifnb1*, *Ifit1*, and *Mx1* mRNA were analyzed by RT-PCR. Data represent mean ± SD from three independent experiments. An unpaired Student's *t*-test was used to determine the statistical significance. $^{**}p < 0.01$. **(H)** p300 knockdown enhances SeV-induced TBK1 and IRF3 phosphorylation. Control (V2) and p300 knockdown L929 cells were left uninfected or infected with the indicated MOIs of SeV. After incubation for 12 h, cell lysates were prepared and analyzed for the indicated proteins by Western blot. Representative blots from one of three independent experiments with similar results are shown.

Figure S3

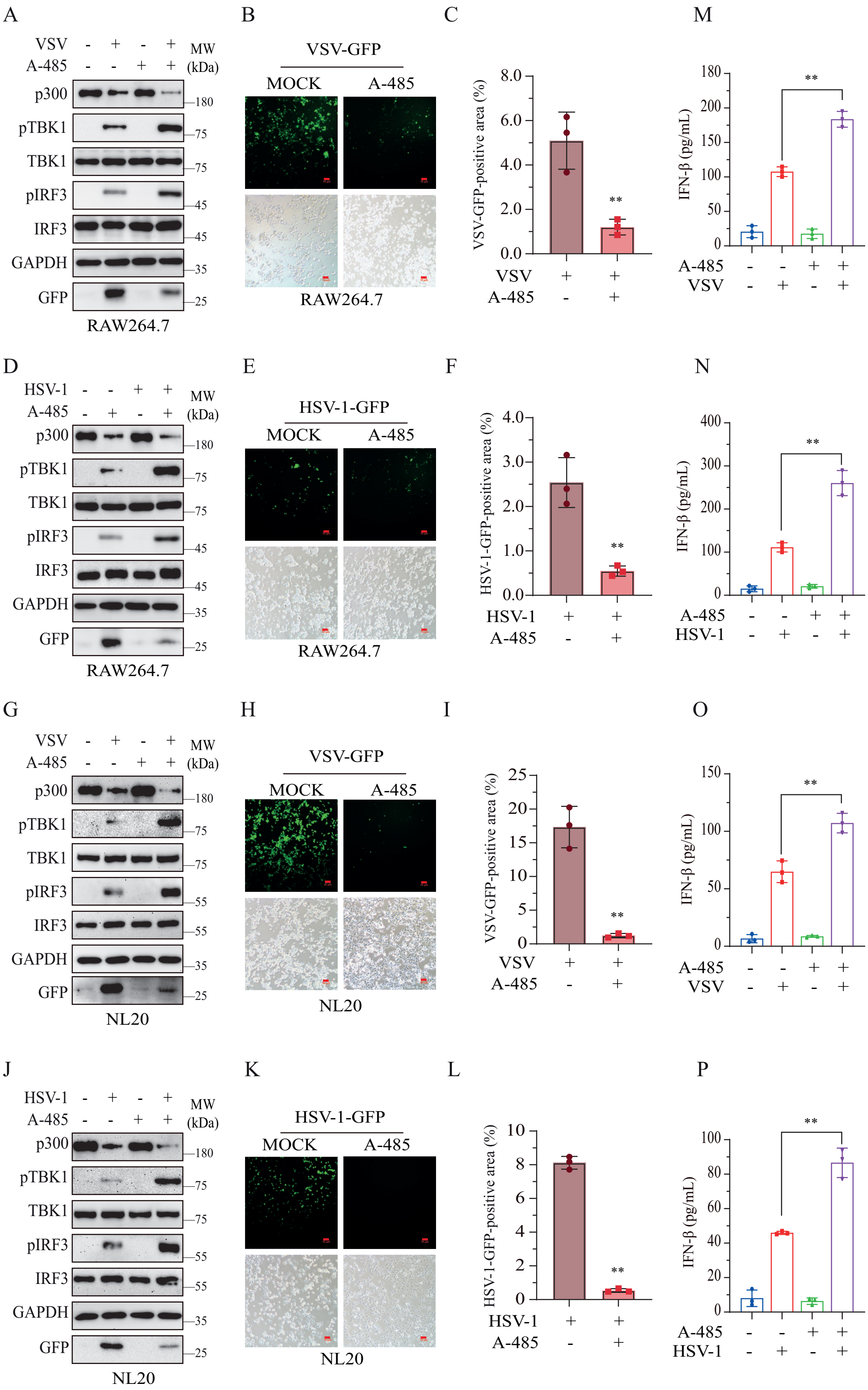

A
VSV
A-485
MW (kDa)
p300
pTBK1
TBK1
pIRF3
IRF3
GAPDH
GFP
RAW264.7
B
VSV-GFP
MOCK
A-485
RAW264.7
C
VSV-GFP-positive area (%)
VSV
A-485
M
IFN-β (pg/mL)
A-485
VSV
D
HSV-1
A-485
RAW264.7
E
HSV-1-GFP
MOCK
A-485
RAW264.7
F
HSV-1-GFP-positive area (%)
N
G
NL20
H
I
O
J
K
L
P

Figure S3. A-485 enhances antiviral responses in RAW264.7 and NL20 cells. (A-F) A-485 enhances TBK1 and IRF3 phosphorylation and inhibits viral replication in RAW264.7 cells. RAW264.7 cells were incubated in the absence or presence of A-485 (10 μM) for 6 h and then left uninfected or infected with VSV or HSV-1 at an MOI of 1 for 12 h. Cell lysates were analyzed for p300 expression, TBK1 and IRF3 phosphorylation, and GFP expression by Western blot **(A & D)**. GFP-positive cells were observed by fluorescence microscopy **(B & E)**, and GFP-positive areas were quantified **(C & F)**. Data represent mean ± SD from three independent experiments. An unpaired Student's *t*-test was used to determine the statistical significance. $^{**}p < 0.01$. **(G-L)** A-485 enhances TBK1 and IRF3 phosphorylation and inhibits viral replication in NL20 cells. NL20 cells were incubated in the absence or presence of A-485 (10 μM) for 6 h and then left uninfected or infected with VSV or HSV-1 at an MOI of 1 for 12 h. Cell lysates were analyzed for p300 expression, TBK1 and IRF3 phosphorylation, and GFP expression by Western blot **(G & J)**. Representative blots from one of three independent experiments with similar results are shown in A, D, G., & J. GFP-positive cells were observed by fluorescence microscopy **(H & K)**, and GFP-positive areas were quantified **(I & L)**. Data are presented as mean ± SD from three randomly selected fields from one of three independent experiments with similar results. **(M-P)** A-485 promotes IFN-β production in RAW264.7 and NL20 cells. RAW264.7 and NL20 cells were incubated in the absence or presence of A-485 (10 μM) for 6 h and then left uninfected or infected with VSV or HSV-1 at an MOI of 1 for 12 h. IFN-β levels in the conditioned media were measured using a mouse IFN-β ELISA kit for RAW264.7 cells and a human IFN-β ELISA kit for NL20 cells. Data represent mean ± SD from three independent experiments An unpaired Student's *t*-test was used to determine the statistical significance. $^{**}p < 0.01$

Figure S4

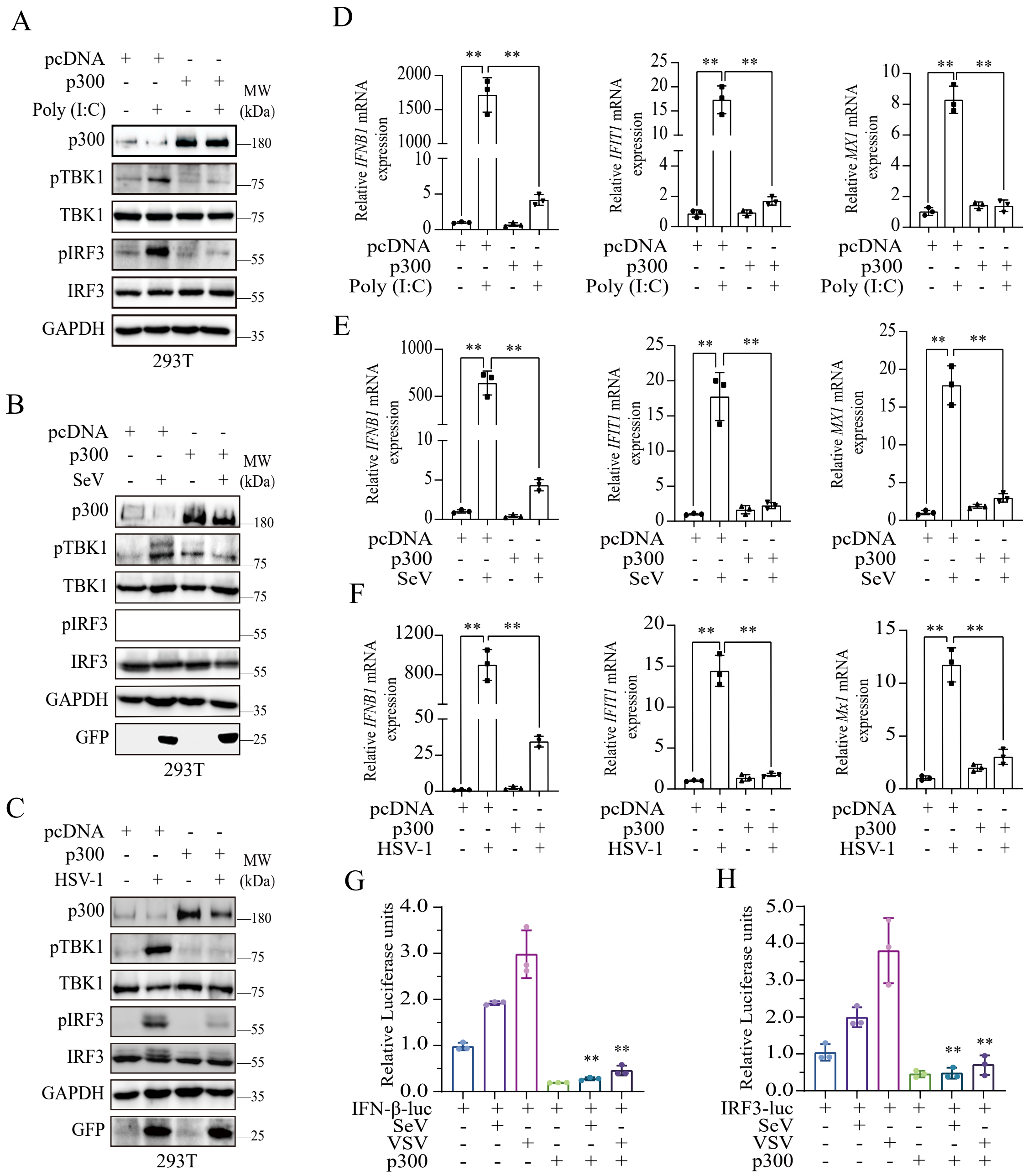

Figure S4. p300 overexpression suppresses TBK1 and IRF3 activation and IFN responses. (**A-C**) p300 overexpression inhibits TBK1 and IRF3 phosphorylation. 293T cells were transfected with pcDNA3.1 or the p300 expression vector. After incubation for 36 h, cells were transfected with poly(I:C) (1 µg) for 8 h (A) or infected with SeV or HSV-1 (1 MOI each) for 12 h (B and C). Cell lysates were analyzed for TBK1 and IRF3 phosphorylation by Western blot. Representative blots from one of three independent experiments with similar results are shown in A-C. (**D-F**) p300 overexpression inhibits IFN-β and ISG mRNA expression. 293T cells were transfected with pcDNA3.1 or the p300 expression vector. After incubation for 36 h, cells were transfected with poly (I:C) (1 µg) for 8 h **(D)** or infected with SeV **(E)** or HSV-1 **(F)** (1 MOI each for 12 h. The levels of *IFNB1*, *ISG56*, and *MX1* mRNA were analyzed by RT-PCR. Data represent mean ± SD from three independent experiments. An unpaired Student's *t*-test was used to determine the statistical significance. $^{**}p < 0.01$. (**G & H**) p300 overexpression suppresses IFN-β promoter and IRF3-responsive reporter activation. 293T cells were co-transfected with pcDNA3.1 or the p300 expression vector together with an IFN-β promoter-driven or IRF3-responsive luciferase reporter plasmid. After incubation for 36 h, cells were left uninfected or infected with SeV or VSV for 12 h. Cell lysates were prepared and analyzed for luciferase activity. Data represent mean ± SD from three independent experiments. An unpaired Student's *t*-test was used to determine the statistical significance. $^{**}p < 0.01$.

Figure S5

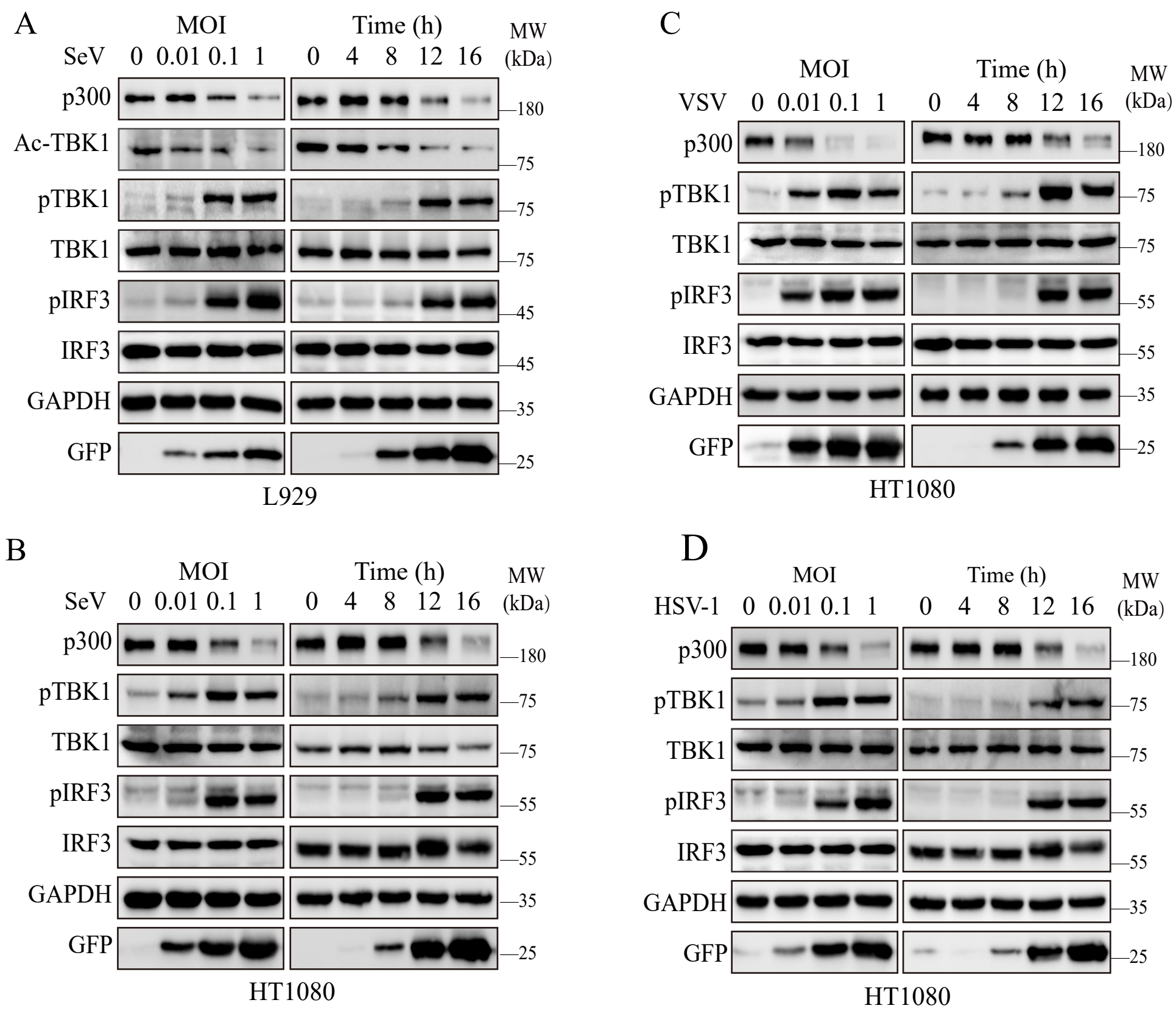

Figure S5. Virus infection reduces p300 expression. (A-D) L929 cells and HT1080 cells were infected with the indicated MOIs of SeV (**A** & **B**), VSV (**C**), or HSV-1 (**D**) for 12 h, or with 1 MOI of these viruses for the indicated lengths of time. Cell lysates were prepared and analyzed by Western blot for p300 expression and TBK1 and IRF3 phosphorylation. TBK1 acetylation (Ac-TBK1) was examined in SeV-infected L929 cells **(A)**. Representative blots from one of three independent experiments with similar results are shown.

Figure S6

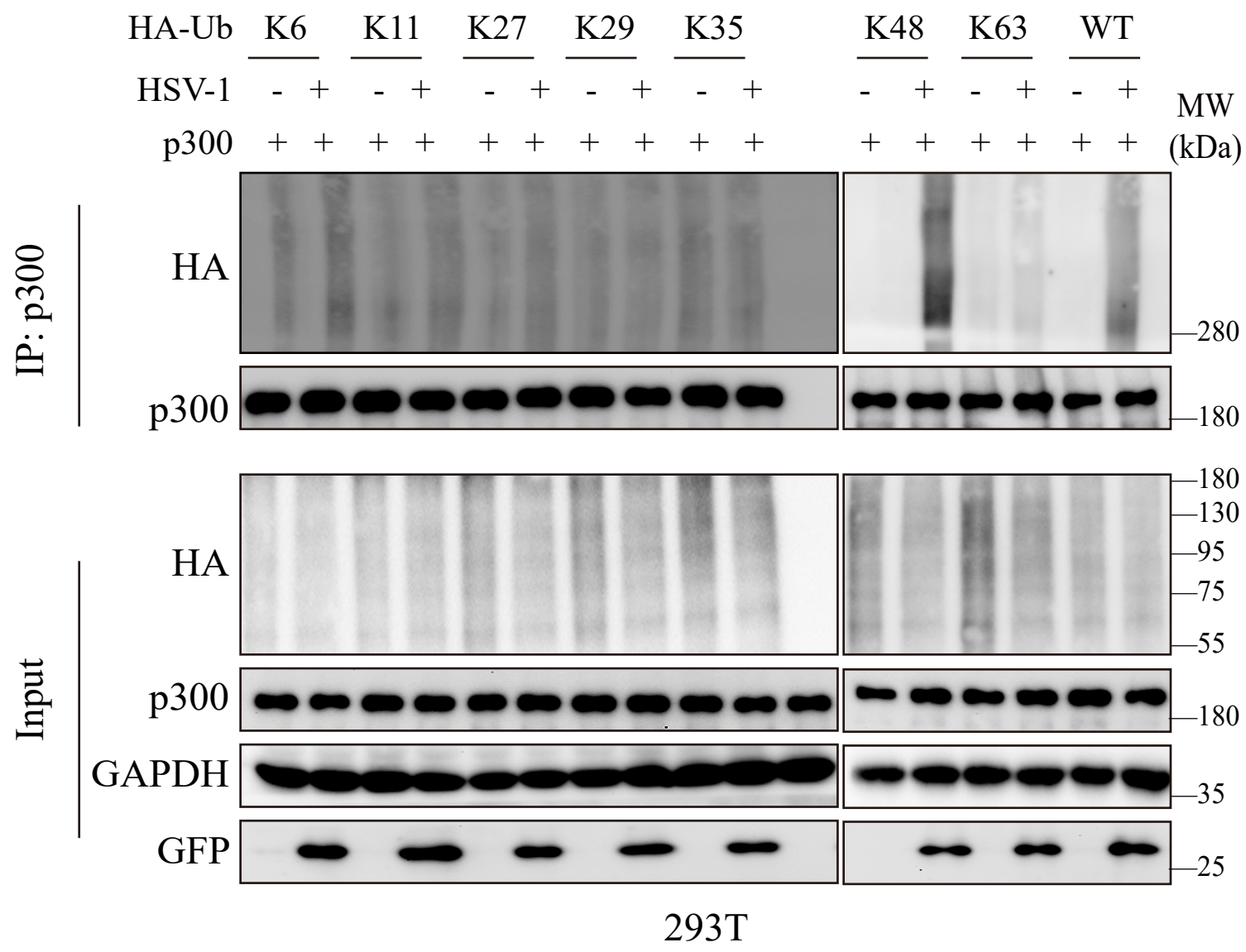

Figure S6. HSV-1 infection promotes K48-linked ubiquitination of p300. (A) 293T cells were co-transfected with plasmids encoding p300 together with HA-Ub-WT or the indicated HA-tagged ubiquitin lysine mutants, including HA-Ub-K6, HA-Ub-K11, HA-Ub-K27, HA-Ub-K29, HA-Ub-K33, HA-Ub-K48, and HA-Ub-K63. After incubation for 36 h, the cells were left uninfected or infected with HSV-1 (1 MOI). After incubation in the presence of MG132 (10 μM) for 12 h, cell lysates were immunoprecipitated with an anti-p300 antibody followed by detection of ubiquitination with an anti-HA mAb. Cell lysates were analyzed for the expression of indicated proteins by Western blot as input controls. Representative blots from one of three independent experiments with similar results are shown.

Figure S7

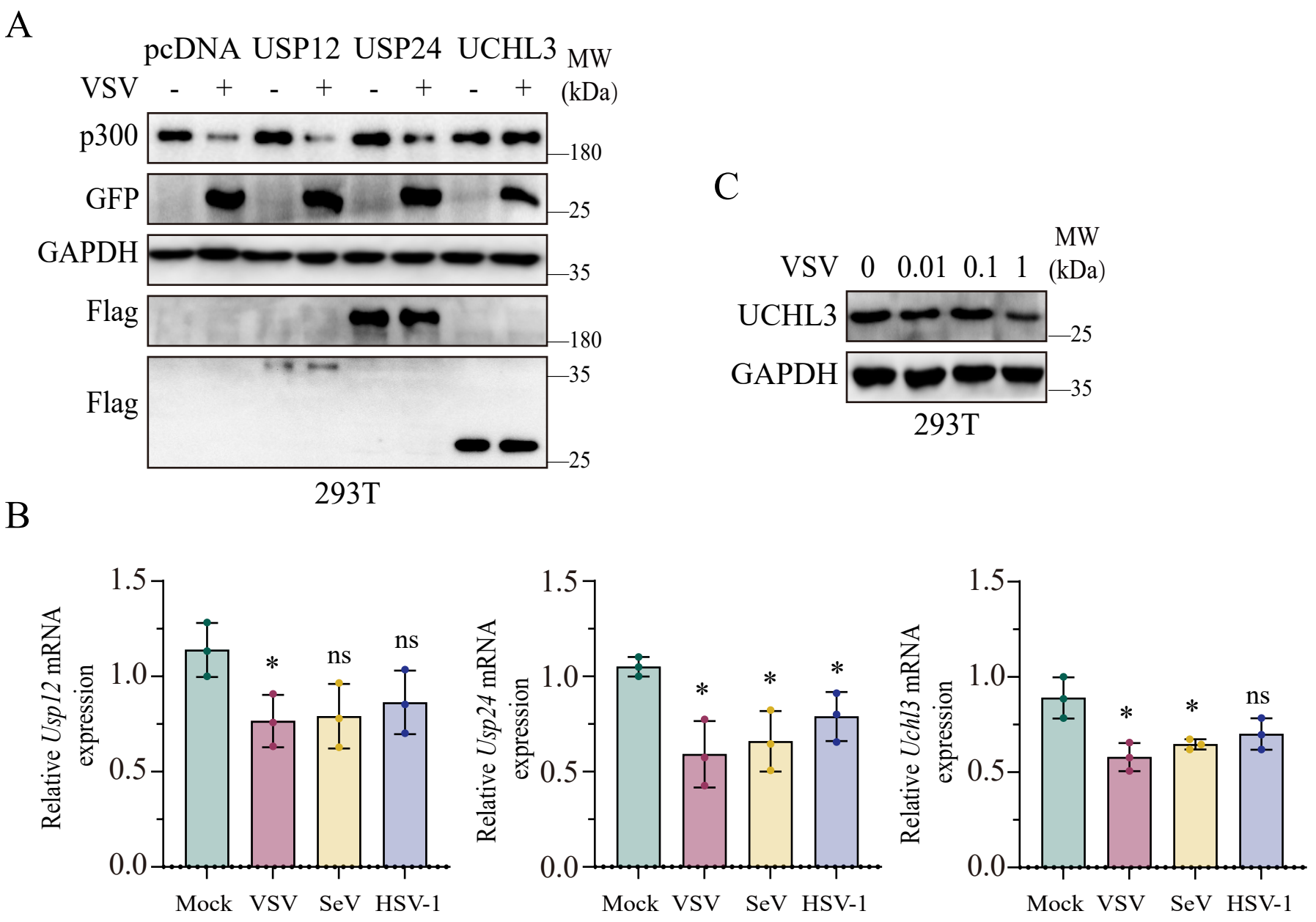

Figure S7. UCHL3 is involved in virus-induced p300 degradation. (**A**) UCHL3 prevents virus-induced p300 degradation. 293T cells were transfected with pcDNA3.1 or expression vectors encoding Flag-tagged USP12, USP24, or UCHL3. After incubation for 36 h, cells were left uninfected or infected with VSV for 12 h. Cell lysates were prepared and analyzed for p300, GFP, Flag, and GAPDH expression by Western blot. Representative blots from one of three independent experiments with similar results are shown. (**B**) Virus infection weakly lowers deubiquitinase mRNA levels. L929 cells were left uninfected or infected with VSV, SeV, or HSV-1 for 12 h. Total RNA was extracted, and the mRNA levels of *Usp12*, *Usp24*, and *Uchl3* were analyzed by RT-PCR. Data represent the mean ± SD of three independent experiments. An unpaired Student's *t*-test was used to determine the statistical significance. *$p < 0.05$; ns, not significant. (**C**) VSV infection weakly reduces UCHL3 expression. 293T cells were infected with VSV at the indicated MOIs for 12 h. Cell lysates were prepared and analyzed by Western blot for UCHL3 and GAPDH expression. Representative blots from one of three independent experiments with similar results are shown.
