# Supplementary material for: p300 Degradation by the p53‐SIAH1 Axis Relieves TBK1 Acetylation to Enhance Innate Antiviral Immunity
Source: Adv Sci (Weinh). 2026 Jun 15;13(50):e76101. doi: 10.1002/advs.76101 (PMC13336735; doi:10.1002/advs.76101)
Supplement: Supplementary file 2 [file ADVS-13-e76101-s001.docx]

**Table S1. The sequences of primers used for gene amplification and cloning**

|  | **Gene** | | **Sequences** |
| --- | --- | --- | --- |
| p300-N | | F: 5’-CCAAGCTGGATGGCCGAGAATGTGGTGG-3’ | |
|  |  | R: 5’-GGTCTTTGTAGTCCTTTTTCTTTGACTGTCCTGGAGCC-3’ | |
| p300-Core | | F: 5’-CCCAAGCTGGATGATTTTCAAACCAGAAGAACTACGAC-3’ | |
|  |  | R: 5’-GTCTTTGTAGTCGTCCTGGCTCTGCGTG-3’ | |
| p300-C | | F: 5’-CCCAAGCTGGATGCGCTTTGTCTACACCTGC-3’ | |
|  |  | R: 5’-CATGGTCTTTGTAGTCGTGTATGTCTAGTGTACTCTGTGAGAGG-3’ | |
| SIAH1 | | F: 5’-CCCAAGCTGGATGTCATCAGTGAGCCCCATC-3’ | |
|  | | R: 5’- CCGTCATGGTCTTTGTAGTCTTTCCATGTGTTGAAATGGCAATCAAAC-3’ | |
| USP12 | | F: 5’-CCCAAGCTGGATGGAAATCCTAATGACAGTCTCCAAATTCG-3’ | |
|  | | R: 5’-CTTTGTAGTCGTCCCGAGACTGATAGAAAAGGATG-3’ | |
| USP24 | | F: 5’-CCCAAGCTGGATGGAATCGGAGGAGGAGCAG-3’- | |
|  | | R: 5’-CTTTGTAGTCGGGATCAACATCATCAAGGTCACTTC-3’ | |
| UCHL3 | | F: 5’-CCCAAGCTGGATGGAGGGTCAACGCTGGC-3’ | |
|  | | R: 5’-GGTCTTTGTAGTCTGCTGCAGAAAGAGCAATCGC-3’ | |
| TBK1-K241R | | F: 5’-GTATAAAATAATTACAGGAAgGCCTTCTGGTG-3’  R: 5’-cTTCCTGTAATTATTTTATACATCACTTCTTTATTC-3’ | |
| TBK1-K692R | | F: 5’-GACTCTTGGTATGAAGAgATTAAAGGAAGAG-3’  R: 5’-cTCTTCATACCAAGAGTCATTTCTACTAATG-3’ | |
| TBK1-N159 | | F: 5’-TCTGATATCGCAGAGCACTTCTAATCATCTGTGGC-3’  R: 5’-GATGGATCTGGAAAGACAGTCAACGTTGCGAAGG-3’ | |
| p300-C160 | | F: 5’-GGCCCGAATTGCCGAGAATGTGGTGGAAC-3’ | |
|  |  | R: 5’-CTCGGGATCCGGTGTATGTCTAGTGTACTCTGTGAGAGG-3’ | |

# **Table S2. Primer sequences for qPCR**

| **Gene** | **Sequences** |
| --- | --- |
| *Ifnb1* | F: 5’- CAGCTCCAAGAAAGGACGAAC-3',  R: 5’- GGCAGTGTAACTCTTCTGCAT-3 |
| *Ifit1 (mISG15)* | F: 5’- TAGCCAACATGTCCTCACAGAC-3’  R: 5’- TCTTCTACCACTGGTTTCATGC-3’ |
| *Mx1 (mouse MX1)* | F: 5’- GACCATAGGGGTCTTGACCAA-3’  R: 5’- AGACTTGCTCTTTCTGAAAAGCC-3 |
| *Usp12* | F: 5’-AACAGCACACCAGACCCAACCT-3’  R:5’-TGTTCCACGTCAACAGAAAGGTC-3’ |
| *Usp24* | F: 5’-GAACTGGGCAGAGGTGTTTG-3’  R: 5’-TGGAGTTTGGCTTGGATTGC-3’ |
| *Uchl3* | F: 5’-CAAACCATCAGCAATGCCTGTGG-3’  R: 5’-GGGCTCATTGATACAGACTCCTC-3’ |
| *Actb* | F: 5’- CATCCGTAAAGACCTCTATGCCAAC-3’  R: 5’- ATGGAGCCACCGATCCACA-3’ |
